# Patient satisfaction with advanced practice physiotherapy internationally: A systematic mixed studies review

**DOI:** 10.1371/journal.pone.0342674

**Published:** 2026-02-11

**Authors:** Chris Davis, Tim Noblet, Jodie Breach, Jai Mistry, Kaitlyn Maddigan, Katie Kowalski, Alison Rushton

**Affiliations:** 1 School of Physical Therapy, Faculty of Health Sciences, Western University, London, Ontario, Canada; 2 Nuffield Health Learning Foundation, Nuffield Health, Surrey, England; 3 Therapies Department, St Georges University Hospitals NHS Foundation Trust, London, England; National Institute of Health and Medical Research: INSERM, FRANCE

## Abstract

**Introduction:**

Advanced practice physiotherapy (APP) is internationally recognised as a higher level of practice involving expert clinical and analytical skills to manage complex patient needs. Patient satisfaction measures how pleased someone is with their care, comprises human and system attributes, and is an indicator of patient experience (quality). Patient satisfaction with APP appears high, but no comprehensive evidence synthesis across settings exists. Objectives were to evaluate patient satisfaction with APP internationally, and evaluate human and system attributes of patient satisfaction with APP.

**Materials and methods:**

Systematic mixed studies review using a parallel-results convergent synthesis design. Key databases and grey literature were searched for studies measuring patient satisfaction with APP across clinical fields from inception to September 9^th^, 2025. Screening, data-extraction, and quality appraisal were completed in parallel by two reviewers. Narrative (quantitative) and thematic (qualitative) syntheses were integrated through discussion, and GRADE/GRADE-CERQual assessed evidence confidence and certainty,

**Results:**

35 high (n=8), moderate (n=16), and low (n=11) quality studies were included. Narrative synthesis found very low certainty evidence for high overall and human attributes of patient satisfaction, and mostly high system attributes of patient satisfaction. Thematic synthesis found moderate-high confidence evidence of human attributes of patient satisfaction (proficient communication and interpersonal skills, credible and competent experts, patient empowerment and self-management, thorough assessments) and moderate confidence evidence of system attributes of patient satisfaction (fast access to specialist care, convenient location and amenities, integrated care).

**Conclusion:**

Human and system attributes drive high patient satisfaction with APP. High-confidence evidence suggests that AP physiotherapists themselves are integral to patient satisfaction, as found across research in other professions. Quantitative evidence certainty is very-low, therefore future high-quality research is needed to guide APP service development.

## Introduction

### Rationale

Advanced practice (AP) is a high level of clinical, leadership, education, and research practice, requiring autonomy and complex decision-making and existing across many professions [1]. Advanced Practice Physiotherapy (APP) evolved from US military settings in the 1970s, into at least 15 fields of practice across 14 countries globally [2,3]. In MSK settings, AP physiotherapists show diagnostic accuracy, triage appropriateness, positively impact patient outcomes, and improve access to care [4]. Furthermore, diagnostic and triage concordance between AP physiotherapists and orthopaedic or spinal surgeons is high, with comparable or greater clinical outcomes observed with APP-led models of care when compared to usual care [5,6]. Various educational pathways to APP exist including residency, fellowship, accredited or non-accredited training courses, and master’s level education, with the latter most widely accepted and likely the most comprehensive across all 4-pillars of AP [1,7]. World Physiotherapy, working officially with the World Health Organisation to represent and further the physiotherapy profession, describes APP as a higher level of practice, requiring distinctly increased clinical and analytical skills, applied to achieve improved patient outcomes and experience, in patients with complex needs [8,9]. Furthermore, World Physiotherapy recognises AP physiotherapists perform conventional physiotherapeutic tasks as well as tasks traditionally completed by other healthcare professionals (e.g., prescribing medications) [4,9]. Building on World Physiotherapy’s description of APP, Tawiah et al. (2025) propose an international definition of APP as “a broad term that refers to expert physiotherapists who employ a higher level of competencies and expertise with additional responsibilities and autonomy to manage complex and challenging health needs of individuals, families, and populations within or beyond their scope of practice. APPs demonstrate competencies as expert clinician, communicator, collaborator, leader, health advocate, scholar, and professional” (p12) [10].

Patient satisfaction is a complex phenomenon reflecting how individuals feel about their care, described as a sense of contentedness or fulfilment, that results from meeting needs and expectations [11–14]. Ng and Luk (2019) suggest patient satisfaction comprises two attributes [15]. Human attributes relate to provider attitude and technical competence, with system attributes relating to accessibility and efficacy. Similar attributes are seen in non-clinical settings, with reference to relational (emotional benefits from social interaction) and functional (service efficiency) dimensions of service quality [16]. Patient satisfaction is measured using questionnaires, patient-reported outcome measures, or experience measures, and importantly is an indicator of patient experience [14,17,18]. Patient experience, defined as “the sum of all interactions, shaped by an organisation’s culture, that influence patient perceptions, across the continuum of care” [19], is a valuable measure of healthcare quality [12,14].

Research investigating patient satisfaction with APP lacks comprehensive analysis and is often a briefly reported secondary objective in systematic reviews investigating models of care. In this research, high patient satisfaction is found across outpatient musculoskeletal (MSK), spinal pain, emergency department, and orthopaedic diagnostic settings [6,20–25]. APP is often rated as equivalent to, or more satisfactory than physician-led care, with the opportunity to spend more time with AP physiotherapists (than with physicians) cited a possible reason [6,22]. However, methodological quality of primary studies within these reviews varies, and heterogeneity exists across patient satisfaction measures. Furthermore, the included primary studies are mostly conducted in MSK fields, use quantitative methodologies, and seldom offer explanations of why a patient was satisfied (or not) with APP. Considering patient satisfaction provides valuable insight into patient experience (a component of healthcare quality), and no comprehensive systematic review synthesising patient satisfaction across settings exists, further investigation is needed.

### Objectives

1) To evaluate patient satisfaction with APP internationally.
2) To evaluate human and system attributes of patient satisfaction with APP.

## Materials and Methods

Reported using Preferred Reporting Items for Systematic review and Meta-Analyses (PRISMA) [26] and the published protocol (PROSPERO: CRD42023443612) [27].

### Design

Systematic mixed studies review (SMSR) with parallel-results convergent design [28]. Objective-1 was addressed quantitatively, and objective-2 was addressed quantitatively and qualitatively. SMSR was selected as mixed evidence allows comprehensive understanding of complex phenomena (patient satisfaction) and because objectives were not measuring effectiveness [29].

### Eligibility criteria

Eligibility criteria (Table 1) were informed by the PICOS framework [32]. Studies were grouped for synthesis according to quantitative and qualitative design.

**Table 1:** Eligibility criteria [27].

Participants	Patients of APP services from countries, territories or jurisdictions with recognised APP roles who can give informed consent. Studies of parent or carer satisfaction of APP services on behalf of a dependent were excluded.
Intervention	APP as described by World Physiotherapy [30] whereby practitioners a) have advanced clinical and analytical skills that influence service improvement and provide clinical leadership, b) have post-registration masters level specialisation (or equivalence), c) deliver safe, competent care to patients with complex needs and d) may be associated with particular occupational titles, in all clinical fields (e.g., Musculoskeletal, orthopaedics, or emergency department).
Comparison	Not applicable.
Outcome	Patient satisfaction. Studies investigating only patient experience were excluded.
Study designs	Primary research of any design (e.g., experimental, qualitative, or mixed methods).
Publication language	English, or papers able to be sufficiently translated into English via Google Translate [31].

APP: Advanced Practice Physiotherapy

### Information sources

MEDLINE (Ovid), Embase, CINAHL, Cochrane, Web of Science, and PEDro databases, grey literature (ProQuest), and trial registers (clinicaltrials.gov and WHO International Clinical Trials Registry Platform) were searched from inception to September 9^th^, 2025. Manual reference list searches and consultations with expert researchers also attempted to identify further studies.

### Search strategy

Search strategies were developed in line with the published protocol, using patient satisfaction, physiotherapy, and advanced practice constructs. An independent specialist librarian refined the MEDLINE (Ovid) search strategy, which was further adapted to meet other database search terms (S1 File).

### Study records

#### Data management.

Covidence, a web-based systematic review software, was used to import citations, remove duplicates, and determine eligibility during screening.

#### Selection process.

**Fig 1:**
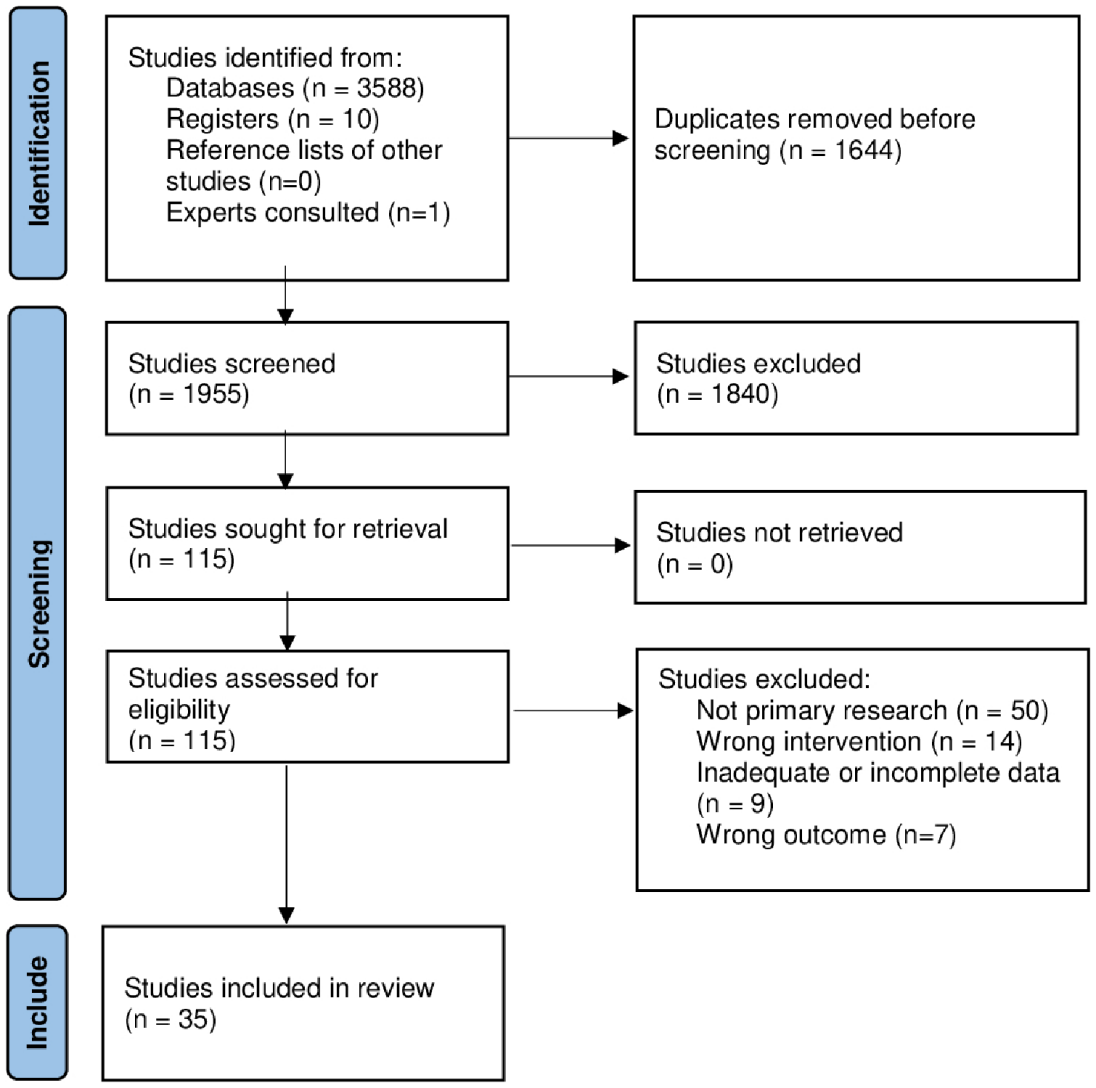
PRISMA flow diagram.

Eligibility criteria instruction was provided before screening to ensure consistency. Two reviewers screened titles and abstracts against eligibility criteria independently. Full texts were obtained and imported into Covidence for articles meeting eligibility criteria or deemed indeterminable. Full texts were screened against eligibility criteria by two reviewers independently. Inter-rater screening agreement was calculated using Cohens-kappa (k). The full selection process is illustrated in the PRISMA flow diagram (Fig 1).

#### Data collection process.

Data were collected by two reviewers independently into a Microsoft Excel form with pre-set data items. Training provided prior to data collection helped maintain quality and consistency. Data from 5 studies were first collected by both reviewers and subsequently compared to ensure consistency. Following full data collection, data were collated with discrepancies resolved through discussion. Authors of one study [33] were contacted twice to address incomplete data, but due to lack of response the study was excluded.

### Data items

Data items were sought in line with the published protocol, including study characteristics, population, intervention, and outcome data.

### Quality assessment

The mixed methods appraisal tool (MMAT) assessed methodological quality of included studies. The MMAT is a valid, reliable, and efficient tool [34,35] developed to appraise methodological quality of five empirical study categories (qualitative, randomised-controlled trial, non-randomised, quantitative descriptive, and mixed methods) eligible for inclusion [36]. Two reviewers familiarised themselves with the MMAT before independently completing quality assessment. A pilot of 5 studies were appraised and discussed to ensure reviewer consistency. The first two MMAT questions ensured the paper was an empirical study [36], then criteria were rated “Yes”, “No”, or “Can’t Tell” depending on information reported in the paper. An amendment to the published protocol involved using thresholds and a summary scale to present MMAT outcomes. These identified high, moderate, and low-quality studies using a percentage of “Yes” responses (0–40% = Low, 60–80% = Moderate, 100% = High). Thresholds were informed by similar research using the MMAT and were included to support narrative synthesis [37,38]. As this SMSR aimed to generate rich understanding of patient satisfaction, studies were not excluded based on methodological quality, as this could have limited insight [39]. Quality assessment was instead used to provide transparency and aid interpretation during thematic synthesis [39,40].

### Synthesis methods

Parallel-results convergent synthesis design, involving separate quantitative and qualitative data syntheses, before integration in the discussion [28]. Meta-analysis or statistical exploration of heterogeneity (e.g., subgroup analysis) was not appropriate.

### Quantitative synthesis

**Fig 2:**
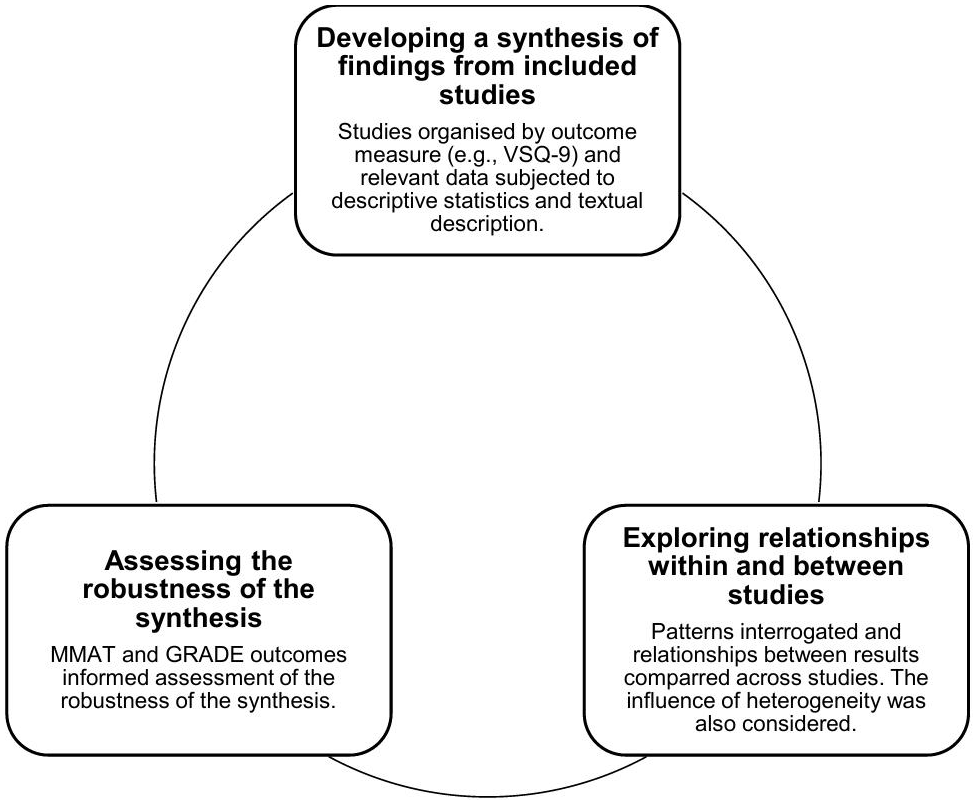
Elements of narrative synthesis [42]. VSQ-9: Visit Specific Satisfaction Questionnaire, MMAT: Mixed Methods Appraisal Tool, GRADE: Grading of Recommendations, Assessment, Development and Evaluation, APP: Advanced Practice Physiotherapy.

To evaluate patient satisfaction with APP internationally (objective 1) and evaluate human and system attributes of patient satisfaction with APP (objective 2), narrative synthesis was conducted [41]. This was chosen due to expected heterogeneity within primary studies and lack of randomised controlled trials (RCT). The methodological foundation of narrative synthesis includes 3 key elements completed iteratively and non-sequentially (Fig 2) [41].

### Qualitative synthesis

**Fig 3:**
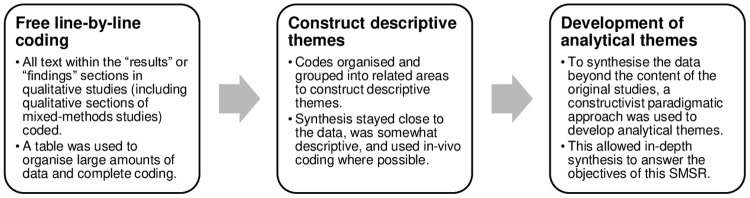
Steps of thematic synthesis [42].

To evaluate human and system attributes of patient satisfaction with APP (objective 2), thematic synthesis was conducted [42]. This was chosen to integrate findings of multiple qualitative or mixed-methods studies and involved three steps (Fig 3) [42].

Two reviewers completed thematic synthesis independently before coming together to discuss analytical themes. This crystallisation stage helped the development of analytical themes that offer rich accounts of patient satisfaction [43]. To increase trustworthiness, reflexivity helped position subjective and intersubjective influences when shaping of analytical themes (S2 File) [44]. Analytical themes were supported by textual descriptions and illustrative quotations in an accompanying table. As analytical themes derived from text within studies’ results sections, differentiation between author and participant quotes were made.

### Certainty assessment

Grading of Recommendations, Assessment, Development and Evaluation (GRADE) assessed certainty of evidence for quantitative syntheses (S3 File) [45]. Most syntheses started as “low” and adaptations to assessments within GRADE domains were made to account for the mostly non-randomized study (NRS) designs. Questions addressing various biases within the MMAT and the overall rating (high to low) informed assessment of the Risk of Bias domain [46]. Indirectness was assessed against how thoroughly studies reported PICOS elements, for example, information provided regarding the AP physiotherapists experience, qualifications, or specific role (intervention) [46]. Without statistical heterogeneity measures, Inconsistency was assessed using descriptive statistics across outcomes within a given measure (e.g., mean averages across VSQ-9 scores). Imprecision was informed by CIs or SE when reported [46]. Total NRS and unpublished papers informed publication bias assessment [46]. Without effectiveness data, rating-up was not appropriate for magnitude of effect or dose-response gradient. All plausible confounding was assessed using study population characteristics considered to introduce confounding (e.g., younger age) [15,47].

GRADE Confidence in Evidence from Reviews of Qualitative Research (GRADE-CERQual) assessed confidence in evidence for qualitative syntheses (S4 File) [48]. Methodological limitations were assessed using the MMAT [49], coherence was assessed considering fit between collected primary study data and review findings [50], adequacy was assessed through data richness and bridging criteria “thoroughness of data collection and interpretation” [51,52], and relevance was assessed by how clearly primary studies reported alignment with eligibility criteria [53]. GRADE-CERQual assessments were presented using a summary of qualitative findings table [54]. Both GRADE and GRADE-CERQual assessments were completed independently by two reviewers, with conflicts resolved through discussion, not requiring a third reviewer.

## Results

### Study selection

Searches yielded 3599 studies. After duplicate removal, 1955 studies underwent title and abstract screening, and 115 studies underwent full-text screening, resulting in 35 studies included (Fig 1) [55–89]. A list of excluded studies from full-text screening is provided (S5 File). Very high reviewer agreement occurred at title and abstract (k = 0. 9) and full-text (k = 0.96) screening. Disagreements were resolved through discussion and did not require third author arbitration.

### Study characteristics

Studies were published between 1999–2025, encompassing RCT (n = 5), quantitative non-randomized (n = 11), quantitative descriptive (n = 3), qualitative (n = 9), and mixed methods (n = 7) designs. Studies originated from Canada (n = 14), Australia (n = 9), United Kingdom (n = 7), Sweden (n = 2), Denmark (n = 1), Republic of Ireland (n = 1), and France (n = 1). All but one study was published [83], and no companion studies were identified. Studies were in MSK/orthopaedic fields, spanning hospital outpatient department (n = 19), emergency department (n = 8), primary care (n = 6), and physiotherapy department (n = 2) settings. Study characteristics are detailed in Table 2.

**Table 2:** Results of individual studies.

Study details and sample	Overall patient satisfaction	Human attributes of patient satisfaction	System attributes of patient satisfaction
Blondin, J et al. (2024) Design: Qualitative, descriptive Setting & specialism Emergency dept (ED) (Canada) – Musculoskeletal Triage Sample size: 11	–	**Qualitative** SSI: Themes/*Sub-theme(s)* Care experience and satisfaction/*Patients felt they received adequate care and were satisfied* Perceived APPs expertise in the ED/*APPs performed thorough assessments and made an accurate diagnosis/APPs gave helpful advice and exercises for musculoskeletal disorders management/APPs were perceived as competent healthcare professionals who could be an alternative to ED physicians*	**Qualitative** SSI: Themes/*Sub-theme(s)* Relevance and benefits of the new model of care/*Timely access was a key benefit of this model*
Bødskov, E B et al. (2022) Design: Mixed methods Setting & specialism Outpatient clinic (Denmark) – Orthopaedic Triage Sample size: 88 (questionnaire), 9 (interview)	VSQ-9: Median (1st and 3rd quartiles) The visit overall 100 (75–100)	**Quantitative** VSQ-9: Median (1st and 3rd quartiles) Explanations during consultation 100 (75–100) Technical skills 100 (75–100) Personal manners 100 (100–100) **Qualitative** SSI: Themes The APP’s health professional profile; Level of information	**Quantitative** VSQ-9: Median (1st and 3rd quartiles) Waiting time for appointment 50 (25–75) Office location 50 (50–75) Getting through by phone 75 (50–75) Waiting time at the office 75 (50–100) Duration of consultation 75 (75–100) **Qualitative** SSI: Themes Expectations; Physical Environment; Practical Issues
Booth, R (2019) Design: Mixed methods Setting & specialism Outpatient clinic (Canada) – Neurosurgery Triage Sample size: 20 (quantitative), 5 (qualitative)	CSQ: Agree/Strongly Agree (%) I am satisfied with the overall service 90% Some things could have been better 20% I am satisfied with the recommendations 90% CSQ: Yes/No Were you satisfied with your consultation? 95% Yes.	**Quantitative** CSQ: Agree/Strongly Agree (%) My examination was very thorough 95% This health care provider was interested in me 85% I felt this health care provider really knew what I was thinking 80% **Qualitative** SSI: Themes Understanding/expectations; Interactions with the APP	**Quantitative** CSQ: Agree/Strongly Agree (%) I wish it had been possible to spend a little more time 35% **Qualitative** SSI: Themes Understanding/expectations; Overall satisfaction
Carey, N et al. (2020) Design: Mixed methods Setting & specialism Private practice, outpatient clinics, and community-based clinics (UK) – Mixed specialisms Sample size: 62	BQ: Agree/Strongly Agree (%) Overall I was satisfied with the consultation from this physiotherapist 95.1% Some things about the consultation with the physiotherapist or podiatrist could have been better (R); 74.2%	**Quantitative** BQ: Agree/Strongly Agree (%) The physiotherapist was very careful to check everything when carrying out my care 96.8% The physiotherapist listened very carefully to what I had to say 91.2% The physiotherapist was interested in me as a person, not just my illness 80.1% I am NOT completely satisfied with the advice received from this physiotherapist (R) 90.3%. I would have liked to have received more information about my medicine from the physiotherapist (R) 13.6%.	**Quantitative** BQ: Agree/Strongly Agree (%) The time I was able to spend with the physiotherapist was a bit too short (R) 74.2% It was easy to make an appointment with the physiotherapist 56.5% There was an acceptable time lapse to obtain an appointment 48.4% It was possible to obtain an appointment on a convenient day or hour 64.5% The waiting time was acceptable 72.5%
Daker-White, G et al. (1999) Design: Randomised-controlled trial Setting & specialism Outpatient clinic (UK) – Orthopaedic Triage Sample size: 192	BQ: First stated number indicates greater satisfaction, mean (SEM) Overall (1–7) 2.7 (0.1)	**Quantitative** BQ: First stated number indicates greater satisfaction, mean (SEM) Staff communication/attitudes (19–95) 43.2 (0.8) Perceived treatment quality (13–65) 28.0 (0.6)	**–**
Desmeules, F et al. (2013) Design: Quantitative non-randomized study, analytical cross-sectional study. Setting & specialism Outpatient clinic (Canada) – Orthopaedic Triage Sample size: 112	VQS-9: Mean (SD) 93.2 (13.5)	–	–
Downie, F et al. (2019) Design: Quantitative descriptive study, survey Setting & specialism General practice, primary care (UK) – Musculoskeletal Assessment Sample size: 75	–	**Quantitative** BQ: Very good (%) Listening to you 96% Explaining tests and treatment 88% Involving you in decisions about your care 89% Treating you with care and concern 91%	**Quantitative** BQ: Very good (%) Giving you enough time 92%
Fennelly, O et al. (2020) Design: Qualitative, descriptive Setting & specialism Outpatient clinics (Republic of Ireland) – Orthopaedic or Rheumatology Triage Sample size: 10	**–**	**Qualitative** SSI: Themes/*Sub-theme(s)* Interpersonal and professional skills/*Confidence in the APP service/Communication skills/Physiotherapist versus doctor*	**Qualitative** SSI: Themes/*Sub-theme(s)* Health service wait times/*More timely access via APP service/Long wait times for other services* Location of ‘specialist’ services/*Transport to hospitals/Hospital experts and facilities*
Gibbs, AJ et al (2020) Design: Mixed methods Setting & specialism Community-based, and Hospital-based outpatient clinic (Australia) – Musculoskeletal Triage Sample size: 91	MedRisk: Mean (SD) Overall, I am completely satisfied with the services I receive from my therapist I would return to this department for future services or care Community OAHKS 4.8 (0.5) Hospital OAHKS 4.7 (0.5)	**Quantitative** MedRisk: Mean (SD) My therapist spends enough time with me My therapist thoroughly explains the treatment(s) I receive My therapist treats me respectfully My therapist answers all my questions, My therapist listens to my concerns My therapist advises me on ways to avoid future problems My therapist gives me detailed instructions regarding my home program Community OAHKS 4.8 (0.5) Hospital OAHKS 4.6 (0.8) **Qualitative** Open Text: Themes/*Sub-theme(s)* Positive patient comments/*Patients value information and advice about osteoarthritis/Therapist time, communication, and attitude/Overall experience.*	**Quantitative** MedRisk: Mean (SD) The reception staff are courteous The process of booking an appointment is appropriate The waiting area is comfortable Community OAHKS 4.8 (0.4) Hospital OAHKS 4.4 (0.6) **Qualitative** Open Text: Themes/*Sub-theme(s)* Positive patient comments/*Waiting time and process.* Suggestions for improvement from patients/*Shorter waiting time/More information about parking, clinic, location/Improved environment; Improve GP awareness*
Gillis, K et al. (2014) Design: Qualitative, descriptive case-study Setting & specialism Outpatient clinic (Canada) – Orthopaedic Triage Sample size: 13	–	**Qualitative** SSI: Themes/*Sub-theme(s)* Perceptions of the ERP model of care/*Time spent with the ERP/Knowledge and competence of the ERP/Education provided by the ERP*	**Qualitative** SSI: Themes/*Sub-theme(s)* Timely access to care Distance as a factor in seeking care Perceptions of the ERP model of care/*Overall effect of the programme*
Goodwin, R et al. (2021) Design: Qualitative, descriptive Setting & specialism General practice, primary care (UK) – Musculoskeletal Assessment Sample size: 14	–	–	**Qualitative** SSI: Patient discourse reflects a positive experience of APP
Gustavsson, L et al. (2023) Design: Randomised-controlled trial Setting & specialism Outpatient clinic (Sweden) – Orthopaedic Triage Sample size: 249	QPP: Median/Q1; Q3 (range from 1 “Completely agree” to 4 “Do not agree at all”. Wanting to visit this clinic in the future 1/1:1	**Quantitative** QPP: Median/Q1; Q3 (range from 1 “Completely agree” to 4 “Do not agree at all”. I received the best possible examination and treatment 1/1:1 Seemed to understand how I experienced my situation 1/1:2	–
Harding, P et al. (2015) Design: Qualitative, descriptive Setting & specialism ED (Australia) – Musculoskeletal Triage Sample size: 25	–	**Qualitative** SSI: Themes The value of personal attributes of staff, including professionalism and communication, was important to participants; Participant confidence in the skills and attributes of APP physiotherapist made them a suitable alternative or preferable to a doctor in these situations	**Qualitative** SSI: Themes Participants were satisfied with the process and level of service provided by the APP; The timing and efficiency of the APP service was better than expected and valued
Harding, P et al. (2018) Design: Mixed Methods Setting & specialism Outpatient clinic (Australia) – Post Arthroplasty Review Sample size: 444	BQ: Satisfied/very satisfied (%) How satisfied were you with your experience being cared for by the AMP? 97%	–	–
Kechichian, A et al. (2024) Design: Qualitative, descriptive Setting & specialism Primary care, general practice (France) – Musculoskeletal (Low Back Pain) Assessment Sample size: 10	–	**Qualitative** SSI: Themes/*Sub-theme(s)* “The physiotherapist offered to give me exercises to do at home to relieve the back pain”/*Positive informed experience was offered for the evaluation and care plan.* “I went there feeling confident”/*Physiotherapists were perceived as competent first-contact practitioners that may replace physicians/Physiotherapists had the competences to make a valid diagnosis, prescribe medication and sick leaves/Physiotherapists gave thorough education and explanations and listened to patients*	**Qualitative** SSI: Themes/*Sub-theme(s)* “The physiotherapist offered to give me exercises to do at home to relieve the back pain”/*Rapid access prevented complications.* “I went there feeling confident”/*The new APP model of care supported interprofessional collaboration*
Kennedy, DM et al. (2010) Design: Quantitative non-randomized study, analytical cross-sectional study Setting & specialism Outpatient clinic (Canada) – Post Arthroplasty Review Sample size: 63	VSQ-9: Mean (95%CI) Visit overall 88.5 (84.4–92.5)	**Quantitative** VSQ-9: Mean (95%CI) Time spent with the healthcare providers 86.9 (82.0–91.8) Answers to your questions 92.1 (88.2–96.0) Explanation of the results of the assessment 92.1 (88.3–95.8) Advice and information about exercise and returning to activities 92.9 (89.2–96.5) Technical skills of the healthcare providers 92.5 (88.6–96.3) Personal manner of the healthcare providers 94.4 (91.4–97.5)	**Quantitative** VSQ-9: Mean Getting through to the Outpatient Clinic by phone Length of time waiting once you arrived) 69.8
Lafrance, S et al (2024) Design: Randomised-controlled trial Setting & specialism Outpatient clinic (Canada) – Spinal Triage Sample size: 106 (single group 52, multiple group 54)	VSQ-9: Mean (SD) Single: 84.5 (19.7); Multiple 92.5 (11.2) VSQ-9: Median (Quartiles) Single: 90.6 (84.4–93.8); Multiple: 100 (90.6–100) MedRisk: Global factors Mean (SD) Single: 3.9 (1.1); Multiple: 4.6 (0.7) MedRisk: Global factors Median (Quartiles) Single: 4 (3–5): Multiple: 5 (4.5–5)	**Quantitative** MedRisk: Internal factors Mean (SD) Single: 3.5 (0.5); Multiple: 3.8 (0.5) MedRisk: Internal factors Median (Quartiles) Single: 3.7 (3.1–3.9); Multiple: 3.8 (3.6–3.9)	**Quantitative** MedRisk: External factors Mean (SD) Single: 4.2 (0.8); Multiple: 4.5 (0.7) MedRisk: External factors Median (Quartiles) Single: 4 (3.7–5); Multiple: 5 (4–5)
Lafrance, S et al (2024) Design: Qualitative descriptive Setting & specialism Outpatient Clinic (Canada) – Spinal Triage Sample Size: 17	–	**Qualitative** SSI: Themes/*Sub-theme(s)* “Advanced practice physiotherapy model of care is a great solution to manage patients with spinal disorders”/*Advanced practice physiotherapists can adequately diagnose and triage patients/Patients benefit from physiotherapy care including education and exercises*	**Qualitative** SSI: Themes/*Sub-theme(s)* “Advanced practice physiotherapy model of care is a great solution to manage patients with spinal disorders”/*Patients are getting the right care at the right time with this newmodel* “Areas for improvement in the advanced practice physiotherapy model of care”/*Patients and physiotherapists report benefits from follow-up sessions/The CareAxis program can still be improved.*
Lowry, V et al (2020) Design: Quantitative non-randomized, analytical cross-sectional study Setting & specialism Outpatient clinic (Canada) – Orthopaedic Triage Sample size: 50	VSQ-9: Mean (SD) Satisfaction was high, with scores of 87.8 (16.6) for the APP.	–	–
Marks, D et al (2016) Design: Randomised-controlled trial Setting & specialism Outpatient clinic (Australia) – Shoulder Injection Sample size: 33	VAS: Mean (SD) 9.64 (0.73)	–	–
Matifat, E et al. (2019) Design: Quantitative non-randomized, analytical cross-sectional study Setting & specialism ED (Canada) – Musculoskeletal Triage Sample size: 91	VSQ-9: Mean (SD) 87.6% (16.3)	–	–
Matifat, E et al. (2025) Design: Quantitative descriptive, cross-sectional study Setting & specialism Outpatient Geriatric Chronic Pain Clinic (Canada) – Assessment & Management Sample size: 65	VSQ-9: Mean (SD) 8.9 (3.6) MedRisk: Mean (SD) 4.1 (0.7)	–	–
McClellan, C M et al. (2006) Design: Quantitative non-randomized, analytical cross-sectional study Setting & specialism ED (UK) – Musculoskeletal Triage Sample size: 351	PSQ: Strongly agree (%) Overall I was satisfied with the treatment received 54.5%	**Quantitative** PSQ: Strongly agree (%) I felt I received good advice and information about my condition 48.4% I was given enough time to ask questions and discuss my condition 45.5% I felt confident the member of staff could deal with my condition 48.4% I felt confident the member of staff would have got a second opinion if necessary 47.2%	–
Morris, J et al. (2015) Design: Mixed methods Setting & specialism ED (Australia) – Musculoskeletal Triage Sample size: 11	–	**Qualitative** SSI: Themes Thoroughness of the physiotherapy examination	**Qualitative** SSI: Themes Not aware initially that they were consulting a PT Lengthy wait times
Murphy, MT & Radovanovic, J (2021) Design: Quantitative non-randomized, analytical cross-sectional study Setting & specialism Outpatient clinic (Australia) – Post Arthroplasty Review Sample size: 50	VSQ-9: Mean (SD) The visit overall 92 (15) All items 89 (14)	**Quantitative** VSQ-9: Mean (SD) Answered questions 92 (14) Explanation of assessment 91 (14) Advice about exercise/activity 89 (16 Technical skills of clinician 91 (14) Manner of clinician 94 (14)	**Quantitative** VSQ-9: Mean (SD) Clinic telephone contact 81 (21) Time waiting at appointment 81 (22) Time in appointment 88 (16)
Razmjou, H et al. (2013) Design: Quantitative non-randomized, analytical cross-sectional study Setting & specialism Outpatient clinic (Canada) – Orthopaedic Triage Sample size: 105	VSQ-9: Mean, Median, Q1/Q3 Overall visit 94, 100, 100/100	**Quantitative** VSQ-9: Mean, Median, Q1/Q3 Answers to questions 93, 100, 88/100 Explanations of the results of the assessment 93, 100, 100/100 Advice and information about exercise and returning to activities 88, 100, 75/100 Technical skills (thoroughness, carefulness, competence) of the health care provider 95, 100, 100/100 Personal manner (courtesy, respect, sensitivity, friendliness) of the health care provider 96, 100, 100/100	**Quantitative** VSQ-9: Mean, Median, Q1/Q3 Time spent with the health care provider 91, 100, 75/100
Resteghini, P (2003) Design: Quantitative descriptive study, survey Setting & specialism Outpatient clinic (UK) – Musculoskeletal Injections Sample size: 69	**Data collection** BQ: Simple point satisfaction scale 28% Very satisfied 66% Quite satisfied 6% Very dissatisfied.	–	–
Robarts, S et al. (2017) Design: Quantitative non-randomized, analytical cross-sectional study Setting & specialism Outpatient clinic (Canada) – Spinal Triage Sample size: 102	VSQ-9: Mean (SD) Visit overall 90.6 (14.8)	**Quantitative** VSQ-9: Mean (SD) Information about APP screening 92.6 (12.9) Explanation of what was done 92.1 (14.9) Information about outcome 86.7 (18.8) Technical skills 91.6 (13.7) Personal manner 95.0 (11.1)	**Quantitative** VSQ-9: Mean (SD) Wait time for appointment 55.1 (39.6) Wait time in clinic 80.8 (25.8) Time spent 92.8 (13.7)
Samsson, KS et al. (2016) Design: Randomised-controlled trial Setting & specialism General practice, primary care (Sweden) – Orthopaedic Triage Sample size: 77	–	**Quantitative** QPP: Mean, Median (IQR) I received the best possible examination and treatment 3.5, 4 (3;4) Seemed to understand how I experienced my situation 3.8, 4, (3;4)	–
Schulz, P et al. (2016) Design: Quantitative non-randomized, analytical cross-sectional study Setting & specialism ED (Australia) – Musculoskeletal Triage Sample size: 117	PSQ: Strongly agree (%) Overall I was satisfied with the treatment received ALBP 51.9% LLSTI 44.6%	**Quantitative** PSQ: Strongly agree (%) I received good advice and information about my condition ALBP 55.5% LLSTI 38.6% I was given enough time to ask questions and discuss my condition ALBP 55.5% LLSTI 42.2% I felt confident the member of staff could deal with my condition ALBP 53.8% LLSTI 43.4% I felt confident the member of staff would have got a second opinion if necessary ALBP 53.8% LLSTI 42.2%	–
Soever, L et al. (2023) Design: Mixed methods Setting & specialism Virtual consultation (Canada) – Musculoskeletal Assessment Sample size: 374	PSQ: Mean 5-Point Likert Scale Response Scores/5 Overall, I am satisfied with my virtual visit S/E 4.7, LB 4.8, H/K 4.7, F/A 4.7.	**Quantitative** PSQ: Mean 5-Point Likert Scale Response Scores/5 I had confidence and trust with the virtual care assessment S/E 4.6, LB N/A, H/K 4.7, F/A 4.7.	**Quantitative** PSQ: Mean 5-Point Likert Scale Response Scores/5 The main reason for my virtual care visit was dealt with to my satisfaction S/E 4.6, LB N/A, H/K 4.7, F/A 4.6 Did you find the video and any resources provided helpful to allow you to better prepare for the virtual care appointment? S/E 4.2, LB N/A, H/K 4.5, F/A 4.5 **Qualitative** Open Text: Themes Importance of personal connection; Preparatory materials key to virtual care success; Patient perspectives on virtual physical examination; Practical advantages of virtual care Invisible in the virtual waiting room; Technical issues.
Taylor, NF et al. (2011) Design: Quantitative non-randomized study, cohort study Setting & specialism ED (Australia) – Musculoskeletal Triage Sample size: 182	BQ: Strongly agree (%) Overall I was satisfied with the treatment I received 85%	**Quantitative** BQ: Strongly agree (%) I felt I received good advice and information about my condition 83% I felt confident the member of staff could deal with my condition 87% I felt confident the member of staff would have got a second opinion if necessary 77%	**Quantitative** BQ: Strongly agree (%) I was given enough time to ask questions and discuss my injury 86%.
Truter, P et al. (2024) Design: Quantitative non-randomized, case-control study Setting & specialism ED (Australia) – Musculoskeletal Triage Sample size: 208	PSQ: Overall, I was satisfied with my treatment experience 97.6% strongly agree or agree	–	–
Vader, K et al. (2022) Design: Qualitative interpretive phenomenology Setting & specialism General practice, primary care (Canada) – Musculoskeletal Assessment Sample size: 18	–	**Qualitative** SSI: Themes/*Sub-theme(s)* Positive Patient Experiences and Perceived Outcomes with the New Model of Care/*Therapeutic alliance between the physiotherapist and patient/The physiotherapist provided comprehensive care/Improved confidence in managing LBP*	**Qualitative** SSI: Themes/*Sub-theme(s)* Enhanced Primary Care Delivery for LBP/Improved access and engagement in physiotherapy care/Improved communication and care integration between the physiotherapist and primary care team Positive Patient Experiences and Perceived Outcomes with the New Model of Care/*Decreased impact of pain on daily life* Challenges Implementing the New Model of Care/*Challenges with prompt access to physiotherapy care/Challenges making the physiotherapist the first contact for LBP*
Wood, L et al (2022) Design: Qualitative descriptive Setting & specialism General practice, primary care (UK) – Musculoskeletal Assessment Sample size: 430	–	**Qualitative** Open Text: Themes Communication; Treatment and assessment; Clinician characteristics	**Qualitative** Open Text: Themes Treatment and assessment; Efficiency; Comparison to GP care

### Quality assessment

Studies were rated as high (n = 8), moderate (n = 15), and low (n = 11) quality (S6 File). Qualitative studies demonstrated highest, and quantitative descriptive studies lowest methodological quality. Quality criteria met the least were assessor blinding to intervention (RCT) and confounders accounted for in design and analysis (quantitative non-randomized).

### Results of individual studies

Key results of individual studies are presented in Table 2 and expanded results of individual studies are available in S7 File.

### Results of syntheses

#### Narrative synthesis: Overall patient satisfaction.

Across twenty-three studies capturing quantitative data, overall patient satisfaction with APP was consistently high, regardless of instrument, study quality, or analytic approach [55,56,58–60,64–66,68–71,73–77,80,81,83,86–88].

- Ten moderate (n = 8) and low (n = 2) quality studies using a modified visit specific satisfaction instrument (VSQ-9, 0 “poor” to 100 “excellent”) showed excellent mean (84.5–94) and/or median (90.6–100) satisfaction [55,60,70,74–77,81,87,88], although the certainty of this finding was rated as very low using GRADE.
- Three moderate (n = 2) and low (n = 1) quality studies using the patient satisfaction questionnaire (PSQ, 5-point Likert scale, strongly agree – strongly disagree) showed 45–85% “strongly agree” with “overall I was satisfied with the treatment received” [58,64,71], although the certainty of this finding was rated as very low using GRADE.
- Three studies of high, moderate, and low quality using the MedRisk patient satisfaction instrument (1 “Strongly Disagree” – 5 “Strongly Agree”), showed high mean overall satisfaction (3.9–4.8) [68,87,88], although the certainty of this finding was rated as very low using GRADE.
- Three moderate (n = 1) and low (n = 2) quality studies using bespoke 5-point Likert scale questionnaires of varied wording, showed high collated positive responses (i.e., both agree and strongly agree) for overall satisfaction (90%−98%) [65,83,86], although the certainty of this finding was rated as very low using GRADE.

Six individual high (n = 1), moderate (n = 2), and low (n = 3) quality studies all showed high overall satisfaction using different outcome measures or analyses (Table 2) [56,59,66,69,73,80].

#### Narrative synthesis: Human attributes of patient satisfaction (HAPS).

Across seventeen studies capturing quantitative data, HAPS with APP were consistently high, regardless of instrument, study quality, or analytic approach [55,58,60–62,64–66,68–71,75,76,80,83,88].

- Five moderate (n = 4) and low (n = 1) quality studies using a modified VSQ-9 instrument (“poor” to 100 “excellent”) and measuring five HAPS, showed excellent (mean, median) satisfaction for explanation during consultation (87–93, 100), technical skills (91–95, 100), personal manner (94–96, 100), answers to questions (92–93, 100), and advice and information about exercise or activity (88–93, 100) [55,60,70,75,76]. The certainty of this finding was rated as very low using GRADE.
- Two moderate quality studies using the Quality from the Patients Perspective questionnaire (QPP, 4-point scale, do not agree at all – completely agree), showed complete agreement to “I received the best possible examination and treatment” and “[the caregiver] seemed to understand how I experienced my situation” [62,66]. The certainty of this finding was rated as very low using GRADE.
- Three moderate (n = 2) and low (n = 1) quality studies using the PSQ (5-point Likert scale, strongly agree – strongly disagree) and measuring four HAPS, showed 39–83% “strongly agreed” with “I felt I received good advice and information about my condition”, 42–86% “strongly agreed” with “I was given enough time to ask questions and discuss my condition”, 43–87% “strongly agreed” with “I felt confident the member of staff could deal with my condition”, and 42–77% “strongly agreed” with “I felt confident the member of staff would have got a second opinion if necessary” [58,64,71]. The certainty of this finding was rated as very low using GRADE.
- Two studies of moderate and low quality used bespoke 5-point Likert scale questionnaires of varied wording to measure five HAPS, reported as collated positive responses (i.e., both agree and strongly agree) [65,83]. Positive responses were high for thoroughness of examination (97%, 95%), listening and understanding (91%, 80%), interest in the patient as a person (80%, 85%), advice received (90%), and amount of information provided about medicines (86%). The certainty of this finding was rated as very low using GRADE.

Five individual studies of high (n = 1), moderate (n = 1), and low (n = 3) quality all reported high scores relating to HAPS using different outcome measures or analyses (Table 2) [61,68,69,80,88].

#### Narrative synthesis: System attributes of patient satisfaction (SAPS).

Across twelve studies capturing quantitative data, SAPS with APP were mostly high, regardless of instrument, study quality or analytic approach [55,60,61,65,68–70,75,76,80,83,88].

- Five moderate (n = 4) and low (n = 1) quality studies using a modified VSQ-9 instrument (0 “poor” to 100 “excellent”) and measuring five SAPS, showed good to excellent (mean, median) satisfaction for waiting time for appointment (55, 50), clinic location (N/A, 50), clinic telephone contact (70–81, 75), wait time in clinic (70–81, 75), and time spent in appointment (88–93, 75–100) [55,60,70,75,76]. The certainty of this finding was rated as very low using GRADE.
- Two studies of moderate and low quality used bespoke 5-point Likert scale questionnaires with varied wording to measure five SAPS^,^ reported as collated positive responses (i.e., both agree and strongly agree) [65,83]. Positive responses were moderate for “it was easy to make an appointment with the physiotherapist”(57%), “there was an acceptable time lapse to obtain an appointment” (48%), “it was possible to obtain an appointment on a convenient day or hour” (65%), and “the waiting time was acceptable” (73%). Furthermore, “time spent with the physiotherapist was too short” presented as 74% “disagree and strongly disagree” in one study, and 35% “agree and strongly agree” in the other. The certainty of this finding was rated as very low using GRADE.

Five individual studies of high (n = 1), moderate (n = 1), and low (n = 3) quality all reported high scores relating to HAPS using different outcome measures or analyses (Table 2) [61,68,69,80,88].

### Thematic synthesis

Fourteen studies of high (n = 6), moderate (n = 4), and low (n = 4) quality captured qualitative data pertaining to patient satisfaction with APP [55,57,63,67,68,72,78–80,82–85,89]. Twelve studies captured HAPS [55,57,63,67,68,72,78,82–85,89], and all studies captured SAPS. Supporting quotes presented in Table 3.

**Table 3:** Supporting quotes for qualitative themes.

Attribute	Theme	Quotes and source
Human	Proficient communication and interpersonal skills	*“[the* AP Physiotherapist*] actually listened to what I was saying, I find that a lot of the time, particularly if you’re older … that people don’t really listen” Patient* [90] *“she explained the whole process, she was excellent, every question I needed answering she answered before I even had to ask” Patient* [67] *“respondents valued good listening skills, and commented that helpful information provided in a clear and understandable way was important” Author* [63] *“participants described how the physiotherapist was genuine, present, respectful, and personable” Author* [72] *“It was really the [APP] who explained how and what I had to do” Patient* [89]
	Credible and competent experts	*“respondents valued a diagnosis and treatment plan, and the opportunity to consult with a knowledgeable specialist or expert in the field of their condition” Author* [63] *“interviewees perceived the* AP Physiotherapist’s *as confident, professional and knowledgeable… and trusted in their professional ability” Author* [82] *“participant confidence in the skills and attributes of [*AP Physiotherapist’s*] made them a suitable alternative or preferable to a doctor in these situations” Author* [78] *“physios probably know more of the sort of anatomy... bone structures and stuff like that... than a GP would, so to me it’s the right person for the role” Patient* [78] *“they considered the* AP Physiotherapist *a competent examiner, with a few patients mentioning that they believed the* AP Physiotherapist *to be just as competent as an Orthopaedic surgeon” Author* [55]
	Patient empowerment and self-management	*“shared‐decision making was reflected as a valuable component of the [*AP Physiotherapist*] session” Author* [63] *“that therapeutic alliance was there, right, so you kind of feel like you’re in charge but [the* AP Physiotherapist *is] there to support you” Patient* [72] *“although most patients did not require any further secondary care interventions, it was important to them that they were involved in the decision, received reassurance and made progress in the health service” Author* [82] *“glad to… know and understand about my condition, learn how to manage the pain, how to do the right exercise, hopefully leading to better health” Patient* [68] *“Lisa was provided with multiple options regarding her management and reported feeling overwhelmed by the options provided as she had expected to be told what to do” Author* [82]
	Thorough assessments	*“participants reported that they valued that the physiotherapist conducted a thorough physical exam was not rushed in their clinical visits” Author* [72] *“she took the time, I think that was definitely a plus, she took the time to examine me, to answer my questions, and I answered her questions… I felt like I was important to her” Patient* [67] *“I felt very confident that I’ve been scrutinized carefully and thoroughly, so, I wasn’t concerned about that” Patient* [83] *“[the* AP Physiotherapist*] took [their] time explaining things, did more tests to um you know to try to fully diagnose what was going on and um I thought that was... encouraging” Patient* [72] *“participants shared that the physiotherapist made them feel like a person (versus a patient or a number)” Author* [72] *“‘I found her really competent, you know, she really tried every range of motion my leg could do, then looked at all my joints and she was careful to see which parts were hurting” Patient* [85]
System	Fast access to specialist care	*“many respondents interpreted the [APP] service as efficient and as providing quick access to specialised care” Author* [63] *“inherent in the patient satisfaction expressed with [APP] services was the speed and ease of access” Author* [79] *“my first impression, it’s been excellent, it’s had me in and out of here way quicker than it would have done if I’d have had to wait for the General Practitioner” Patient* [79] *“it saves wait times for a lot of situations and I think the diagnosis by the physiotherapist is certainly good, and thorough” Patient* [83] *“Participants reported being received shortly after calling for an appointment, either on the same day or on the next day” Author* [84]
	Convenient location and amenities	*“despite the convenience of the primary care centre, the hospital appeared to be the setting of choice for patients, as it was considered more specialised with access to experts and investigations” Author* [82] *“I was a little leery about being so far away from home [the local hospital] doesn’t seem so far away from home as being in… the city. I was uneasy about that” Patient* [67] *“what was good for me was living so far away and not having to travel to see the doctor [for follow-ups] because I think that’s detrimental to my condition” Patient* [67] *“all patients expressed satisfaction with the physical environment of the clinic, such as the waiting room” Author* [55] *“when patients expressed being less satisfied, it concerned practical matters such as… a need for free parking” Author* [55]
	Integrated care	*“patient participants described how this model of care allowed the physiotherapist and primary care team to easily share information and provide more integrated primary care for low back pain” Author* [72] *“several participants commented on the flow and smooth processes of the service, which appears to be an important factor in participants feeling satisfied” Author* [78] *“when patients expressed being less satisfied, it concerned practical matters such as… lack of collaboration between their general practitioner and the clinic concerning exchange of information” Author* [55] *“the [*AP Physiotherapist*] would like me to have a scan and the waiting list publicly was 2 years … so I obviously went privately” Patient* [82] *“potentially related to this were the frustrations described by some participants with respect to delays in onward referrals” Author* [63]

AP: Advanced Practice, GP: General Practitioner, APP: Advanced Practice Physiotherapy

### Thematic synthesis: Human attributes of patient satisfaction (HAPS)

Four analytical themes emerged pertaining to HAPS: proficient communication and interpersonal skills, credible and competent experts, patient empowerment and self-management, and thorough assessments.

*Proficient communication and interpersonal skills* [55,63,67,68,72,78,82–85,89]*:* Patients appreciate how AP physiotherapists proficiently use active listening, and clear, simple, effective communication during consultations. Patients are satisfied with education provided by AP physiotherapists and being kept informed of key progress updates relevant to their care. Patients value AP physiotherapists’ interpersonal and relational skills, their approachability, empathy, and ability to create an environment that fosters therapeutic alliance. High confidence evidence for proficient communication and interpersonal skills (GRADE-CERQual).

*Credible and competent experts* [55,63,67,68,72,78,82–85,89]: Patients recognise AP physiotherapists as credible, knowledgeable experts. This results in confidence and trust in their professional ability and competence, with APP being seen as equivalent, or preferable to doctor-led care in some settings. High confidence evidence for credible and competent experts (GRADE-CERQual).

*Patient empowerment and self-management* [55,63,68,72,82–85]*:* Patients appreciate AP physiotherapists’ use of shared decision making and being empowered to steer their own recovery. Reassurance and encouragement provided by AP physiotherapists gives patients confidence to self-manage their condition, providing a sense of control. Conversely, some patients felt overwhelmed when too involved in decision-making regarding their care, perhaps preferring a more autocratic style. High confidence evidence for patient empowerment and self-management (GRADE-CERQual).

*Thorough assessments* [57,63,67,72,83–85,89]*:* Patients feel AP physiotherapists conduct thorough assessments, valuing the opportunity to spend time and ask questions regarding their care. Time taken to conduct thorough assessments also perhaps contributes to patients experiencing person-centred care. Moderate confidence evidence for thorough assessments (GRADE-CERQual).

### Thematic synthesis: System attributes of patient satisfaction

Three analytical themes emerged from synthesis of qualitative data pertaining to SAPS: fast access to specialist care, convenient location and amenities, and integrated care.

*Fast access to specialist care* [55,57,63,67,68,72,78–80,82–85,89]*:* Patients perceive APP to speed up access to specialist care. Patients perceive this as reducing time to diagnosis and receiving the right treatment for their condition promptly. Furthermore, patients appreciate shorter than expected wait times, seeing APP as an accessible, effective and efficient service. Moderate confidence evidence for fast access to specialist care (GRADE-CERQual).

*Convenient location and amenities* [55,63,67,68,80,82,83,89]*:* The location of APP services is important to patients, with mixed preference between hospital, urban, community, or rural settings. A key factor driving satisfaction with location appears to be travel distance or time, with stronger preference for convenient services closer to patients’ homes. The amenities (or lack of) and physical environment of APP services is also important to patients (e.g., parking facilities, waiting room comfort). Moderate confidence evidence for convenient location and amenities (GRADE-CERQual).

*Integrated care* [55,63,68,72,78,80,82–84,89]*:* APP is positively received when effectively integrated into the wider care pathway. This integration, for example through co-located clinics in community or hospital settings, is seen to facilitate communication between AP physiotherapists and doctors. Conversely, patients are dissatisfied when integration does not exist and get frustrated with long wait-times for referrals into other services (e.g., imaging) following APP. Moderate confidence evidence for integrated care (GRADE-CERQual).

## Discussion

This SMSR found patient satisfaction with APP internationally is high, based on quantitative data assessed using GRADE, which indicated very-low certainty evidence. Quantitative synthesis showed HAPS were also high (very-low certainty evidence, GRADE), supported by qualitative themes indicating AP physiotherapists use proficient communication and interpersonal skills, are credible and competent experts, drive patient empowerment and self-management, and conduct thorough assessments (moderate to high confidence evidence, GRADE-CERQual). Quantitative synthesis showed SAPS were mostly high (very-low certainty evidence, GRADE), supported by qualitative themes suggesting APP enables fast access to specialist care, with convenient locations and amenities, and through integrated care (moderate confidence evidence, GRADE-CERQual).

### Convergence between syntheses

Convergence was observed when quantitative and qualitative syntheses substantiated one another, offering triangulation and strengthening the conviction of findings. HAPS showed strong convergence between syntheses, especially the theme “proficient communication and interpersonal skills” strongly represented quantitatively with high scores in personal manner, explanation during consultations, and listening attributes. This finding is likely crucial to unlocking many other drivers of patient satisfaction with APP. Communication that purposefully involves the patient, demonstrates active listening, is patient-centred, and uses verbal and nonverbal techniques is known to build therapeutic alliance and drive engagement in physiotherapy encounters [91]. The AP physiotherapist’s ability to employ a broad range of these advanced communication skills, modified to account for individual patient needs or preferences, and to communicate complex information in simple terms, undoubtedly contributes to driving patient satisfaction. The theme “credible and competent experts” was reflected in high scores for technical skills and patients’ confidence in AP physiotherapists’ ability, and the theme “thorough assessments” in high scores for thoroughness of examination and patients feeling they had time to ask questions. Both these findings relate to the concept of trust between patients and AP physiotherapists. Trust is fundamental to effective relationships between healthcare providers and patients, facilitating positive interactions, rapport, and can have direct therapeutic effects, all of which are important for driving satisfaction [92]. Convergence in SAPS also occurred, but to a slightly lesser extent. The theme “fast access to specialist care” was reflected in high scores for acceptable time to obtaining an appointment, and waiting time in clinic, and the theme “convenient location and amenities” in high scores for clinic location, and ease of making an appointment at convenient times. These findings centre around the dimensions of healthcare accessibility, availability, and acceptability, all of which are intimately connected to patient satisfaction [93]. When healthcare services provide the right care, to address individualised needs, at the right time, patient expectations are met or surpassed, and satisfaction is likely to occur. The ability of APP to integrate at various stages of the care pathway, especially in primary care, triage, or diagnostic roles, evidently improves timely access and drives satisfaction with services. Two qualitative themes not reflected in the quantitative data were “patient empowerment and self-management” and “integrated care”. This is likely because patient satisfaction measures in the primary studies did not evaluate these concepts, either due to limitations in the measurement tools or perhaps because these phenomena are better explored qualitatively.

High convergence across quantitative and qualitative syntheses suggests both HAPS and SAPS influence overall satisfaction with APP. However, HAPS were more apparent in this review. More qualitative themes emerged, more studies captured quantitative data, and more syntheses occurred, resulting in higher overall convergence. The prominence of HAPS suggests AP physiotherapists themselves are integral to driving patient satisfaction.

### Context

Our findings align with World Physiotherapy’s definition of APP as a “level of practice, functions, responsibilities, activities and capabilities” [8], as these features closely mirror HAPS. It is AP physiotherapists’ communication proficiency, expertise, or thoroughness that drives satisfaction, reflecting an advanced *level* of practice rather than skills associated with further *scope* of practice (e.g., injection therapy). Congruence between this APP definition and what drives satisfaction with APP from the patient’s perspective, strengthens the credibility of this definition.

High patient satisfaction with AP also exists across other professions [94–96]. High patient satisfaction occurs in cancer, emergency, and critical-care advanced nursing practice (ANP), often with equivalent or greater satisfaction from nurse-led, compared to physician-led care [97,98]. Although not a focus area for our review, equivalence and preference to physician-led care emerged from thematic synthesis (*credible and competent experts*), strengthening the case for alternative care models. Likewise, high patient satisfaction exists in ANP-led memory assessment clinics, driven by the nurse’s sensitivity, empathy, and knowledge [99], as found with APP in our review (*proficient communication and interpersonal skills, credible and competent experts*). These attributes help form therapeutic alliance from assessment, through to multimodal care, a journey AP physiotherapists are well equipped to own. In advanced practice radiography (APR), patient satisfaction is high in therapeutic settings, driven by comprehensive consultations, patient empowerment, time available, opportunities for questions, being well informed, and treatment efficacy [100,101]. These HAPS with APR are congruent with our findings, showing AP physiotherapists deliver patient-centred and personalised care.

Our review supports the conceptual distinction between HAPS and SAPS as proposed by Ng and Luk (2019) [15]. For example, provider attitude was reflected as a human attribute in our review through the AP physiotherapists’ personal manner, interest in the patient as a person, and interpersonal skills. However, some attributes were less clearly delineated. Efficacy was considered a human attribute if patients felt confident APP *could* manage their condition (i.e., future-tense), as satisfaction depended on perceptions of AP physiotherapists’ competence and capability. Conversely, if patients felt APP *did* improve their problem (i.e., past-tense), perhaps by reducing time to diagnosis and receiving treatment, this was a system attribute. Although not an attribute, another concept less delineated was time. If patients felt AP physiotherapists took time to listen and conduct thorough assessments (regardless of appointment duration), it was a human attribute. Whereas if patients were satisfied with the appointment duration, it was a system attribute. The blurred boundaries identified in our review support the notion that HAPS and SAPS are dynamic and contextual in nature and should be interpreted accordingly [15]. Findings from this SMSR also reinforce Donabedian’s model of healthcare quality, illustrating relationships between healthcare structure (SAPS), process (HAPS), and outcome (Patient Satisfaction) [102,103]. In this theoretical framework, Structure relates to the physical and organisational aspects of healthcare, encompassing rational performance, accessibility, culture, and perceived efficacy of services. Process relates to methods and procedures of care delivery, encompassing interactions with healthcare providers, their attitudes or technical competence, and emotions evoked from healthcare services. Outcome relates to the effects of healthcare on patients, which in this instance is patient satisfaction.

### Limitations

This SMSR was limited by the modest methodological quality of the included quantitative studies and by the application of GRADE to predominantly non‑randomised designs, which is a recognised challenge [104]. Additionally, GRADE assessments were informed by MMAT outcomes, which penalise lack of blinding, even though blinding in physiotherapy research is often difficult or impossible [105]. GRADE also rates non-randomised designs down, even though observational research may provide highly relevant, real‑world evidence for outcomes such as patient satisfaction. Together, these factors may have contributed to the conservative “very low” certainty ratings across quantitative syntheses, despite the consistency of findings across the primary studies.

All included studies were mostly in diagnostic MSK or orthopaedic settings. AP standards state advanced practitioners should provide interventions like therapy, lifestyle advice, and care [105], and patient satisfaction with these aspects of APP is not reflected in this review. Furthermore, clinical encounters in MSK and orthopaedics are typically outpatient-based and as attributes of patient satisfaction will be relevant to this context, caution is encouraged when considering applicability to inpatient-based APP.

Lastly, although not a methodological limitation, studies originated from all but one World Physiotherapy region (South America) with recognised APP roles [2]. While our findings are largely representative of APP internationally, caution is encouraged when considering generalisability to South America. This under-representation may be due to only Peru having recognised APP roles demonstrated in the literature to date, the emerging nature of physiotherapy across the region, and/or Brazil producing most South American physiotherapy research [106].

### Clinical implications

The human or relational aspect of an APP patient encounter matters to patients. Strong communication skills like active listening, clear explanations, and providing advice all appear to be important to patient satisfaction. AP physiotherapists should be supported to develop these interpersonal and relational skills alongside their development of technical skills and expertise. Patient empowerment also appears to be integral to patient satisfaction with APP. Therefore, to drive patient satisfaction with APP, services should adopt principles of personalised care like shared decision-making, supported self-management, and social prescribing. Lastly, integrating APP services within the wider healthcare system is essential to drive satisfaction. Therefore, when implementing or improving APP services, careful consideration to how APP seamlessly integrates with its co-dependent services should be a priority.

### Research implications

High methodological quality studies using prospective or randomised designs, and with consideration of confounding variables, are needed to improve evidence certainty. Future research should investigate relationships between HAPS and SAPS and explore if compensatory mechanisms exist, as suggested in service quality settings outside healthcare [16,107]. A comprehensive review of APP patient preferences, values, and expectations is also needed, as already explored across other levels of physiotherapy practice [108–110]. Patient satisfaction is closely connected to these concepts, and understanding what matters to APP patients (across the care pathway) could improve patient centredness. Lastly, deeper exploration into patient experience of APP (as a key component of healthcare quality) and how patient satisfaction is situated within it, is needed. Future research exploring these constructs could provide valuable insights into patient-centred care and inform future APP service quality improvement. Central to all future research in this field is the need for a standardized, valid, and reliable tool that is specifically designed to measure patient satisfaction with APP. This would allow more precise measurement of patient satisfaction and benchmarking of APP services across healthcare sectors and settings.

## Conclusion

Overall patient satisfaction with APP internationally is high, driven by both HAPS and SAPS. Despite the very-low certainty of this evidence, there is moderate to high confidence that AP physiotherapists drive high patient satisfaction as proficient communicators and credible experts, who conduct thorough assessments and drive patient empowerment. There is also moderate confidence in high patient satisfaction being achieved through APP services enabling fast access to specialist care, at convenient locations, whilst situated within integrated care systems.

### Key Points

#### Findings.

Overall patient satisfaction with APP internationally is high, driven by human and system attributes.

#### Implications.

AP physiotherapists are key to patient satisfaction. Ensuring AP physiotherapists are supported to develop interpersonal and communication skills, alongside technical skills, is essential to drive patient satisfaction.

#### Caution.

Methodological quality of quantitative studies was low, no relevant studies from South America were identified, and findings relate to MSK and Orthopaedic settings only.

## Supporting information

S1 File Search strategies. (DOCX)

S2 File Reflexivity statements. (DOCX)

S3 File GRADE Summary of Findings (SoF) table. (DOCX)

S4 File GRADE CERQual evidence profile. (DOCX)

S5 File List of excluded studies. (DOCX)

S6 File Quality assessment. (DOCX)

S7 File Expanded individual studies table. (DOCX)

S1 Checklist PRISMA 2020 checklist. (DOCX)
