# Supplementary material for: Patient satisfaction with advanced practice physiotherapy internationally: A systematic mixed studies review
Source: PLoS One. 2026 Feb 11;21(2):e0342674. doi: 10.1371/journal.pone.0342674 (PMC12893546; doi:10.1371/journal.pone.0342674)
Supplement: S2 File — (DOCX) [file pone.0342674.s002.docx]

**Supplementary file 2. Reflexivity statement**

Lead Author

The lead author of this systematic mixed studies review (SMSR) is male, living and working in England (UK), and is completing doctoral research relevant to this review. This research forms a key part of his thesis, exploring patient experience of advanced practice physiotherapy.

Although he has been a physiotherapist since 2007 and has completed a masters degree in Sports and Exercise Medicine, he currently works mostly non-clinically and has never held an “advanced practice physiotherapist (APP)” title. Clinically he has worked predominantly in musculoskeletal, and his current role involves educational leadership across allied healthcare professions in a UK-based healthcare organisation. This degree of separation from advanced practice physiotherapy may help him approach this research somewhat impartially.

Having lived and worked in the UK, he has a comprehensive knowledge of the national health service (NHS), an organisation that has largely helped shape advanced practice through necessity for innovative workforce models. He is therefore closely aware of the value advanced practitioners have on services, through working alongside APPs in NHS settings and being a recipient of AP led services. This close understanding of advanced practice should help him interrogate research in this field through both professional and personal lenses.
