# Supplementary material for: Patient satisfaction with advanced practice physiotherapy internationally: A systematic mixed studies review
Source: PLoS One. 2026 Feb 11;21(2):e0342674. doi: 10.1371/journal.pone.0342674 (PMC12893546; doi:10.1371/journal.pone.0342674)
Supplement: S3 File — (DOCX) [file pone.0342674.s003.docx]

**Supplementary file 3. GRADE Summary of Findings (SoF) table**

| **Section** | **Measure** | **Contributing studies and design(s)** | **GRADE domains** | | | | | | | | **Certainty of evidence** | **Comments** |
| --- | --- | --- | --- | --- | --- | --- | --- | --- | --- | --- | --- | --- |
|  |  |  | **Risk of Bias (RoB)** | **Inconsistency** | **Indirectness** | **Imprecision** | **Publication Bias (PB)** | **Magnitude of effect** | **Dose-response gradient** | **All plausible confounding** |  |  |
| Overall satisfaction | VSQ-9 | ^1,4,7–11,13–15^ Quantitative non-randomized, Randomized controlled trial, Quantitative descriptive. | Serious MMAT: moderate (8) and low (2) quality. *Confounding poorly accounted for across studies* | No concerns | Serious concerns. *Lack of detail provided for APP characteristics* | No concerns | No concerns | N/a | N/a | N/a | **Very low** for excellent overall satisfaction measured using the VSQ-9 | Started low (study design) and rated down for RoB and indirectness |
|  | PSQ | ^12,17,18^ Quantitative non-randomized | Serious concerns. MMAT: 2 moderate, 1 low quality study. *Confounding poorly accounted for across studies and concerns regarding sample representative of target population.* | Serious concerns. *Variable range of outcomes* | No concerns | Unclear. *CIs not reported* | No concerns | N/a | N/a | N/a | **Very low** for high overall satisfaction measured using the PSQ | Started at low (study design) and rated down for RoB and inconsistency. |
|  | MedRisk | ^5,8,10^ Randomized controlled trial, Quantitative descriptive. Quantitative non-randomized | Serious concerns. MMAT: 1 moderate, 1 low quality. *Concerns regarding attrition and performance biases.* | No concerns | Serious concerns. *Lack of detail provided for APP characteristics* | No concerns | No concerns | N/a | N/a | N/a | **Very low** for high overall satisfaction measured using the MedRisk | Started at low (study design) and rated down for RoB and indirectness |
|  | Bespoke satisfaction questionnaires | ^2,3,19^  Quantitative non-randomized | Serious concerns. MMAT: 1 moderate, 2 low quality. *Confounding poorly accounted for across studies and concerns regarding attrition bias.* | No concerns | No concerns | Unclear. *CIs not reported* | No concerns | N/a | N/a | N/a | **Very low** for high overall satisfaction measured using bespoke satisfaction questionnaires | Started low (study design) and rated down for RoB. |
| Human attributes | VSQ-9 | ^1,7,13–15^ Quantitative non-randomized | Serious concerns. MMAT: 4 moderate, 1 low quality. *Confounding poorly accounted for across studies* | No concerns | No concerns | No concerns | No concerns | N/a | N/a | N/a | **Very low** for excellent human attributes of patient satisfaction measured using the VSQ-9 | Started at low (study design) and rated down for RoB. |
|  | QPP | ^6,16^ Randomized controlled trial | Serious concerns. MMAT: 2 moderate quality. *Lack of blinding and concerns regarding attrition bias across studies* | No concerns | No concerns | No concerns | Serious concerns. *Only RCT’s included.* | N/a | N/a | N/a | **Very Low** for high human attributes of patient satisfaction measured using the QPP | Started at high (study design) and rated down for RoB and PB. |
|  | PSQ | ^12,17,18^ Quantitative non-randomized | Serious concerns. MMAT: 2 moderate, 1 low quality study. *Confounding poorly accounted for across studies and concerns regarding sample representative of target population.* | Serious concerns. *Variable range of outcomes* | No concerns | Unclear. *CIs not reported* | No concerns | N/a | N/a | N/a | **Very low** for moderate to high human attributes of patient satisfaction measured using the PSQ | Started at low (study design) and rated down for RoB. |
|  | Bespoke satisfaction questionnaires | ^2,3^ Quantitative non-randomized, Randomized controlled trial | Serious concerns. MMAT 1 moderate, 1 low quality. *Confounding poorly accounted for across studies* | No concerns | Serious concerns. *Lack of detail provided for APP characteristics* | Unclear. *CIs not reported* | No concerns | N/a | N/a | N/a | **Very low** for high human attributes of patient satisfaction measured using bespoke satisfaction questionnaires | Started low (study design) and rated down for RoB and indirectness |
| System attributes | VSQ-9 | ^1,7,13–15^ Quantitative non-randomized | Serious concerns. MMAT: 4 moderate, 1 low quality. *Confounding poorly accounted for across studies* | No concerns | No concerns | No concerns | No concerns | N/a | N/a | N/a | **Very low** for good to excellent system attributes of patient satisfaction measured using the VSQ-9 | Started at low (study design) and rated down for RoB. |
|  | Bespoke satisfaction questionnaires | ^2,3^ Quantitative non-randomized | Serious concerns. MMAT 1 moderate, 1 low quality. *Confounding poorly accounted for across studies* | No concerns | Serious concerns. *Lack of detail provided for APP characteristics* | Unclear. *CIs not reported* | No concerns | N/a | N/a | N/a | **Very low** for high system attributes of patient satisfaction measured using bespoke satisfaction questionnaires | Started low (study design) and rated down for RoB and indirectness |

GRADE: Grading of Recommendations, Assessment, Development and Evaluation, VSQ-9: Visit Specific Satisfaction Questionnaire, PSQ: Patient Satisfaction Questionnaire, QPP: Quality from the Patients Perspective, N/A: Not Applicable, CI: Confidence Interval

**References**

1. Bak Bodskov E, Palmhoj Nielsen C, Ramer Mikkelsen L, Martin Klebe T, Terp Hoybye M, Norgaard Madsen M. High Patient Satisfaction with Examination by Advanced Practice Physiotherapists in an Orthopaedic Outpatient Shoulder Clinic: A Cross-Sectional Study Using Quantitative and Qualitative Methods. *Physiother Can*. 2022;74(4):342-352. doi:https://dx.doi.org/10.3138/ptc-2021-0043

2. Booth R. *An Advanced Practice Physiotherapy Spine Triage Service for Adults with Neck and Back Pain: A Feasibility Study*. 2019. https://www.lib.uwo.ca/cgi-bin/ezpauthn.cgi?url=http://search.proquest.com/dissertations-theses/advanced-practice-physiotherapy-spine-triage/docview/2535906865/se-2?accountid=15115

3. Carey N, Edwards J, Otter S, et al. A comparative case study of prescribing and non-prescribing physiotherapists and podiatrists. *BMC Health Serv Res*. 2020;20(1). doi:10.1186/s12913-020-05918-8

4. Desmeules F, Toliopoulos P, Roy JS, et al. Validation of an advanced practice physiotherapy model of care in an orthopaedic outpatient clinic. *BMC Musculoskelet Disord*. 2013;14(100968565):162. doi:https://dx.doi.org/10.1186/1471-2474-14-162

5. Gibbs AJ, Taylor NF, Hau R, et al. Osteoarthritis Hip and Knee Service (OAHKS) in a community health setting compared to the hospital setting: A feasibility study for a new care pathway. *Musculoskelet Sci Pract*. 2020;49(101692753):102167. doi:https://dx.doi.org/10.1016/j.msksp.2020.102167

6. Gustavsson L, Mohaddes M, Samsson K, Beischer S. No major difference in perceived quality of care in patients with hip or knee osteoarthritis assessed in a physical therapy-led triage compared with standard care: a randomized controlled trial. *BMC Musculoskelet Disord*. 2023;24(1):530. doi:https://dx.doi.org/10.1186/s12891-023-06659-5

7. Kennedy DM, Robarts S, Woodhouse L. Patients are satisfied with advanced practice physiotherapists in a role traditionally performed by orthopaedic surgeons. *Physiother Can*. 2010;62(4):298-305. doi:https://dx.doi.org/10.3138/physio.62.4.298

8. Lafrance S, Santaguida C, Perreault K, et al. Is One Enough? The Effectiveness of a Single Session of Education and Exercise Compared to Multiple Sessions of a Multimodal Physiotherapy Intervention for Adults With Spinal Disorders in an Advanced Practice P hys iot her apy Model of Care: A Randomized Controlled Trial. *Journal of Orthopaedic and Sports Physical Therapy*. 2024;54(10):634-646. doi:10.2519/jospt.2024.12618

9. Lowry V, Bass A, Lavigne P, et al. Physiotherapists’ ability to diagnose and manage shoulder disorders in an outpatient orthopedic clinic: results from a concordance study. *J Shoulder Elbow Surg*. 2020;29(8):1564-1572. doi:https://dx.doi.org/10.1016/j.jse.2019.11.030

10. Matifat E, Dubé F, Perreault K, et al. Evaluation of a New Advanced Physiotherapy Practice Model of Care for Patients in a Geriatric Pain Management Clinic: A Prospective Observational Study. *Phys Occup Ther Geriatr*. Published online March 10, 2025:1-14. doi:10.1080/02703181.2025.2473920

11. Matifat E, Perreault K, Roy JS, et al. Concordance between physiotherapists and physicians for care of patients with musculoskeletal disorders presenting to the emergency department. *BMC Emerg Med*. 2019;19(1):67. doi:https://dx.doi.org/10.1186/s12873-019-0277-7

12. McClellan CM, Greenwood R, Benger JR. Effect of an extended scope physiotherapy service on patient satisfaction and the outcome of soft tissue injuries in an adult emergency department. *Emerg Med J*. 2006;23(5):384-387.

13. Murphy MT, Radovanovic J. Patient satisfaction with physiotherapists is not inferior to surgeons in an arthroplasty review clinic: non-inferiority study of an expanded scope model of care. *Aust Health Rev*. 2021;45(1):104-109. doi:https://dx.doi.org/10.1071/AH19217

14. Razmjou H, Robarts S, Kennedy D, McKnight C, Macleod AM, Holtby R. Evaluation of an advanced-practice physical therapist in a specialty shoulder clinic: diagnostic agreement and effect on wait times. *Physiother Can*. 2013;65(1):46-55. doi:https://dx.doi.org/10.3138/ptc.2011-56

15. Robarts S, Stratford P, Kennedy D, Malcolm B, Finkelstein J. Evaluation of an advanced-practice physiotherapist in triaging patients with lumbar spine pain: surgeon-physiotherapist level of agreement and patient satisfaction. *Can J Surg*. 2017;60(4):266-272.

16. Samsson KS, Bernhardsson S, Larsson MEH. Perceived quality of physiotherapist-led orthopaedic triage compared with standard practice in primary care: a randomised controlled trial. *BMC Musculoskelet Disord*. 2016;17(100968565):257. doi:https://dx.doi.org/10.1186/s12891-016-1112-x

17. Schulz P, Prescott J, Shifman J, Fiore J, Holland A, Harding P. Comparing patient outcomes for care delivered by advanced musculoskeletal physiotherapists with other health professionals in the emergency department—A pilot study. *Australasian Emergency Nursing Journal*. 2016;19(4):198-202. doi:10.1016/j.aenj.2016.06.001

18. Taylor NF, Norman E, Roddy L, Tang C, Pagram A, Hearn K. Primary contact physiotherapy in emergency departments can reduce length of stay for patients with peripheral musculoskeletal injuries compared with secondary contact physiotherapy: a prospective non-randomised controlled trial. *Physiotherapy*. 2011;97(2):107-114. doi:10.1016/j.physio.2010.08.011

19. Truter P, Flanagan P, Waller R, et al. Short waits, happy patients and expert care, moving basic musculoskeletal care from the emergency department to a physiotherapist‐led diversion pathway. *Emergency Medicine Australasia*. Published online April 15, 2024. doi:10.1111/1742-6723.14416
