# Supplementary material for: Patient satisfaction with advanced practice physiotherapy internationally: A systematic mixed studies review
Source: PLoS One. 2026 Feb 11;21(2):e0342674. doi: 10.1371/journal.pone.0342674 (PMC12893546; doi:10.1371/journal.pone.0342674)
Supplement: S4 File — (DOCX) [file pone.0342674.s004.docx]

**S4 File. GRADE CERQual evidence profile**

| **Section** | **Review finding and contributing studies** | **GRADE CERQual domains** | | | | **Confidence of evidence** | **Explanation of CERQual assessment** |
| --- | --- | --- | --- | --- | --- | --- | --- |
|  |  | **Methodological limitations** | **Coherence** | **Adequacy of data** | **Relevance** |  |  |
| Human attributes | Proficient communication and interpersonal skills^1–6,8–10,13,14^ | No or very minor concerns | No or very minor concerns | No or very minor concerns | Minor concerns. *4/11 studies did not provide APP characteristics* | **High confidence** in finding “proficient communication and interpersonal skills” | Only minor concerns regarding relevance due to intervention (APP) not clearly defined |
|  | Credible and competent experts ^1–6,8–10,13,14^ | No or very minor concerns | No or very minor concerns | No or very minor concerns | Minor concerns. *4/11 studies did not provide APP characteristics* | **High confidence** in finding “credible and competent experts” | Only minor concerns regarding relevance due to intervention (APP) not clearly defined |
|  | Patient empowerment and self-management ^1–5,9,13,14^ | No or very minor concerns | No or very minor concerns | No or very minor concerns | Minor concerns. 3/9 studies did not provide APP characteristics | **High confidence** in finding “patient empowerment and self-management” | Only minor concerns regarding relevance due to intervention (APP) not clearly defined |
|  | Thorough assessments ^2,3,6,9–11,13,14^ | Minor concerns. 3 *moderate and 1 low quality (MMAT)* | No or very minor concerns | Minor concerns. *1 study lacked thoroughness of data collection and interpretation* | Minor concerns. *3/8 studies did not provide APP characteristics* | **Moderate confidence** in finding “thorough assessments” | Concerns with moderate/low methodological quality, adequacy of data (lacking thoroughness), and relevance due to intervention (APP) not clearly defined |
| System attributes | Fast access to specialist care ^1–14^ | Minor concerns. 4 *moderate and 3 low quality (MMAT)* | No or very minor concerns | No or very minor concerns | Minor concerns. *5/14 studies did not provide APP characteristics* | **Moderate confidence** in finding “fast access to specialist care” | Concerns with moderate/low methodological quality and relevance due to intervention (APP) not clearly defined |
|  | Convenient location and amenities ^1,3–6,10,12,14^ | Moderate concerns. 4 *moderate and 1 low quality (MMAT)* | No or very minor concerns | No or very minor concerns | Minor concerns. *4/8 studies did not provide APP characteristics* | **Moderate confidence** in finding “convenient location and amenities” | Concerns with moderate/low methodological quality and relevance due to intervention (APP) not clearly defined |
|  | Integrated care ^1,3–5,8–10,12–14^ | Moderate concerns. 4 *moderate and 2 low quality (MMAT)* | No or very minor concerns | No or very minor concerns | Minor concerns. *4/10 studies did not provide APP characteristics* | **Moderate confidence** in finding “integrated care” | Concerns with moderate/low methodological quality and relevance due to intervention (APP) not clearly defined |

GRADE-CERQual: Grading of Recommendations, Assessment, Development and Evaluation-Confidence in Evidence from Reviews of Qualitative Research, APP: Advanced Practice Physiotherapy, MMAT: Mixed Methods Appraisal Tool.

**References**

1. Bak Bodskov E, Palmhoj Nielsen C, Ramer Mikkelsen L, Martin Klebe T, Terp Hoybye M, Norgaard Madsen M. High Patient Satisfaction with Examination by Advanced Practice Physiotherapists in an Orthopaedic Outpatient Shoulder Clinic: A Cross-Sectional Study Using Quantitative and Qualitative Methods. *Physiother Can*. 2022;74(4):342-352. doi:https://dx.doi.org/10.3138/ptc-2021-0043

2. Blondin J, Desmeules F, Matifat E, Kechichian A. Patients presenting with musculoskeletal disorders in the emergency department: A qualitative study of their experiences when cared by advanced practice physiotherapists in the province of Québec. *Musculoskeletal Care*. 2024;22(3). doi:10.1002/msc.1914

3. Booth R. *An Advanced Practice Physiotherapy Spine Triage Service for Adults with Neck and Back Pain: A Feasibility Study*. 2019. https://www.lib.uwo.ca/cgi-bin/ezpauthn.cgi?url=http://search.proquest.com/dissertations-theses/advanced-practice-physiotherapy-spine-triage/docview/2535906865/se-2?accountid=15115

4. Fennelly O, Blake C, FitzGerald O, et al. Advanced musculoskeletal physiotherapy practice: The patient journey and experience. *Musculoskelet Sci Pract*. 2020;45(101692753):102077. doi:https://dx.doi.org/10.1016/j.msksp.2019.102077

5. Gibbs AJ, Taylor NF, Hau R, et al. Osteoarthritis Hip and Knee Service (OAHKS) in a community health setting compared to the hospital setting: A feasibility study for a new care pathway. *Musculoskelet Sci Pract*. 2020;49(101692753):102167. doi:https://dx.doi.org/10.1016/j.msksp.2020.102167

6. Gillis K, Augruso A, Coe T, et al. Physiotherapy extended-role practitioner for individuals with hip and knee arthritis: patient perspectives of a rural/urban partnership. *Physiother Can*. 2014;66(1):25-32. doi:https://dx.doi.org/10.3138/ptc.2012-55

7. Goodwin R, Moffatt F, Hendrick P, Stynes S, Bishop A, Logan P. Evaluation of the First Contact Physiotherapy (FCP) model of primary care: a qualitative insight. *Physiotherapy*. 2021;113(p8c, 0401223):209-216. doi:https://dx.doi.org/10.1016/j.physio.2021.08.003

8. Harding P, Prescott J, Block L, O’Flynn AM, Burge AT. Patient experience of expanded-scope-of-practice musculoskeletal physiotherapy in the emergency department: a qualitative study. *Aust Health Rev*. 2015;39(3):283-289. doi:https://dx.doi.org/10.1071/AH14207

9. Kechichian A, Pommier D, Druart L, Lowry V, Pinsault N, Desmeules F. “Cooperation between physicians and physios fosters trust you know”: a qualitative study exploring patients’ experience with first-contact physiotherapy for low back pain in French primary care. *BMC Primary Care*. 2024;25(1):69. doi:10.1186/s12875-024-02302-x

10. Lafrance S, Marien L, Desmeules F, Cunningham C, Santaguida C, Lowry V. Patients and Advanced Practice Physiotherapists’ Experiences and Perceptions in a Specialized Spine Model of Care: A Qualitative Study. *Physiotherapy Canada*. Published online November 4, 2024. doi:10.3138/ptc-2024-0023

11. Morris J, Vine K, Grimmer K. Evaluation of performance quality of an advanced scope physiotherapy role in a hospital emergency department. *Patient Relat Outcome Meas*. 2015;6(101551170):191-203. doi:https://dx.doi.org/10.2147/PROM.S75173

12. Soever L, Courchene A, Correale M, et al. Patient-Reported Experiences of Musculoskeletal Virtual Care Delivered by Advanced Practice Physiotherapists. *PHYSIOTHERAPY CANADA*. Published online 2023. doi:10.3138/ptc-2022-0084

13. Vader K, Donnelly C, French SD, et al. Implementing a new physiotherapist-led primary care model for low back pain: a qualitative study of patient and primary care team perspectives. *BMC primary care*. 2022;23(1):201. doi:https://dx.doi.org/10.1186/s12875-022-01817-5

14. Wood L, Bishop A, Goodwin R, Stynes S. Patient satisfaction with the first contact physiotherapy service: Results from the national evaluation survey. *Musculoskeletal Care*. 2022;20(2):363-370. doi:https://dx.doi.org/10.1002/msc.1599
