# Supplementary material for: Patient satisfaction with advanced practice physiotherapy internationally: A systematic mixed studies review
Source: PLoS One. 2026 Feb 11;21(2):e0342674. doi: 10.1371/journal.pone.0342674 (PMC12893546; doi:10.1371/journal.pone.0342674)
Supplement: S5 File — (DOCX) [file pone.0342674.s005.docx]

**S5 File. List of excluded studies at full text screening**

**Not primary research**

1. Pilot of a rheumatology extended scope practitioner to improve the triage process and assess patients with non-inflammatory conditions. Fish S.J.; Macphie E.; Madan A. Rheumatology (United Kingdom) / 2018;57(Supplement 3):iii158 DOI: [10.1093/rheumatology/key075.490](https://dx.doi.org/10.1093/rheumatology/key075.490)
2. Gynaecology physiotherapy screening clinics improve access to care and health outcomes for women on gynaecology outpatient waiting lists. Edwards H.; Greitschus J.; Nucifora J.; Weekes C.; Kuys S.; Sam S. Neurourology and Urodynamics / 2017;36(Supplement 3):S175-S176
3. Service valuation: Patient perceptions of virtual consultations versus advanced physiotherapy practitioner perceptions. Grififths J.; Davies R.; Williams H. Physiotherapy (United Kingdom) / 2022;114(Supplement 1):e106 DOI: [10.1016/j.physio.2021.12.053](https://dx.doi.org/10.1016/j.physio.2021.12.053)
4. A multidisciplinary telemedicine program for identification of spondyloarthritis in medically underserviced communities. Hawke C.; Passalent L.; Haroon N.; Inman R.D.; Rampersaud Y.R. Arthritis and Rheumatology / 2017;69(Supplement 10):
5. Implementation of a first contact physiotherapy service within GP practices in North East Wales. A four-year evaluation. Doran A. Physiotherapy (United Kingdom) / 2021;113(Supplement 1):e137-e138 DOI: [10.1016/j.physio.2021.10.129](https://dx.doi.org/10.1016/j.physio.2021.10.129)
6. Advanced physiotherapy in primary care. Part of the solution for a growing crisis? Hensman-Crook A. Physiotherapy (United Kingdom) / 2017;103(Supplement 1):e112 DOI: [10.1016/j.physio.2017.11.159](https://dx.doi.org/10.1016/j.physio.2017.11.159)
7. A 6-month evaluation of a clinical specialist physiotherapist's role in a fracture clinic. Moloney A; Dolan M; Shinnick L; Murphy M; Wallace L Physiotherapy Ireland 06// 2009;30(1):8-15
8. The extended scope physiotherapist in orthopaedic out-patients - an audit. Pearse, E O; Maclean, A; Ricketts, D M. Annals of the Royal College of Surgeons of England / 2006;88(7):653-5
9. Advanced practitioner physiotherapist as 1st point of contact in a GP cluster in Lanarkshire. Ross J.; McGowan S.; Wightman N. Physiotherapy (United Kingdom) / 2019;105(Supplement 1):e96 DOI: [10.1016/j.physio.2018.11.071](https://dx.doi.org/10.1016/j.physio.2018.11.071)
10. From implementation to integration-physiotherapist-led model changes the face of orthopaedics in Australia: The journey from Vancouver to Amsterdam. Smith D.; Raymer M. Physiotherapy (United Kingdom) / 2011;97(SUPPL. 1):eS1597-eS1598 DOI: [10.1016/j.physio.2011.04.003](https://dx.doi.org/10.1016/j.physio.2011.04.003)
11. Advanced physiotherapy practitioner consultation as an alternative to GP consultation for patients with musculoskeletal conditions. Crerar D. Physiotherapy (United Kingdom) / 2020;107(Supplement 1):e149-e150 DOI: [10.1016/j.physio.2020.03.218](https://dx.doi.org/10.1016/j.physio.2020.03.218)
12. A physiotherapy led minor injuries unit during the covid pandemic. McDonough A.; Troedel M.; Dawson T.; Jeavons K.; Marsden L. Physiotherapy (United Kingdom) / 2022;114(Supplement 1):e125 DOI: [10.1016/j.physio.2021.12.078](https://dx.doi.org/10.1016/j.physio.2021.12.078)
13. Evaluation results utilizing advanced practice physiotherapist in rural Canadian family practice for patients with hip and knee arthritis. Soever L.; Currie S.; Gross A.; Mihu E.; Kaszas Z.; Backstein D.; Safir O.; Jenkins L. Physiotherapy (United Kingdom) / 2011;97(SUPPL. 1):eS1601 DOI: [10.1016/j.physio.2011.04.003](https://dx.doi.org/10.1016/j.physio.2011.04.003)
14. The value of non-medical prescribing by physiotherapists for patients, the service and the profession. Ramaswamy B. Physiotherapy (United Kingdom) / 2011;97(SUPPL. 1):eS1579 DOI: [10.1016/j.physio.2011.04.003](https://dx.doi.org/10.1016/j.physio.2011.04.003)
15. The unique challenges and opportunities of delivering a First Contact Physiotherapy (FCP) Service to remote island communities in NHS Highland. Gillies M.; Arnaud J. Physiotherapy (United Kingdom) / 2021;113(Supplement 1):e160-e161 DOI: [10.1016/j.physio.2021.10.163](https://dx.doi.org/10.1016/j.physio.2021.10.163)
16. Using advanced practice physiotherapists in a rheumatology service to reduce waiting times and improve appropriateness of patient care. Monahan K.; Sayce J. Physiotherapy (United Kingdom) / 2022;114(Supplement 1):e39-e40 DOI: [10.1016/j.physio.2021.12.289](https://dx.doi.org/10.1016/j.physio.2021.12.289)
17. East Lothian community advanced practitioner project. Dey S. Physiotherapy (United Kingdom) / 2022;114(Supplement 1):e175-e176 DOI: [10.1016/j.physio.2021.12.148](https://dx.doi.org/10.1016/j.physio.2021.12.148)
18. Improved access and targeting of musculoskeletal services in northwest Wales: targeted early access to musculoskeletal services (TEAMS) programme. Maddison, Peter; Jones, Jeremy; Breslin, Anne; Barton, Craig; Fleur, Joyce; Lewis, Rhian; McSweeney, Luke; Norgain, Carys; Smith, Sara; Thomas, Carolyn; Tillson, Chris. BMJ (Clinical research ed.) / 2004;329(7478):1325-7
19. Patient satisfaction and outcomes of MSK pain patients accessing advanced physiotherapy practitioner in primary care; a service evaluation. Morley H.; Ker K. Physiotherapy (United Kingdom) / 2019;105(Supplement 1):e164-e165 DOI: [10.1016/j.physio.2018.11.168](https://dx.doi.org/10.1016/j.physio.2018.11.168)
20. A specialist fitness for work service: Advanced practice physiotherapist working as an FCP. Chetty, Laran. Frontline (20454910) 11// 2020;26(11):30-31
21. Review of the Effectiveness of a Consultant Physiotherapy-Led Musculoskeletal Interface Team: A Welsh Experience. Candy, Elizabeth; Haworth-Booth, Sam; Knight-Davis, Mark. Musculoskeletal care / 2016;14(3):185-91 DOI: [10.1002/msc.1122](https://dx.doi.org/10.1002/msc.1122)
22. Implementation of a virtual spinal clinic (VSC) for patients with acute spinal pathology providing timely management and reducing face-to-face follow-up. Abbott C.; Watt T.; Oxborrow N.; Siddique I.; Verma R.; Angus M. Physiotherapy (United Kingdom) / 2020;107(Supplement 1):e168-e169 DOI: [10.1016/j.physio.2020.03.247](https://dx.doi.org/10.1016/j.physio.2020.03.247)
23. Evaluation of an advanced practice physiotherapist role in rheumatology. Farrer C. Arthritis and Rheumatism / 2013;65(SUPPL. 10):S893 DOI: [10.1002/art.38216](https://dx.doi.org/10.1002/art.38216)
24. GIRFT IN ACTION: ADVANCED PRACTICE PHYSIOTHERAPIST AND ADVANCED PRACTICE NURSE EXPERTISE PROVIDES VALUABLE TRIAGE AND ASSESSMENT CLINICS FOR NON-URGENT RHEUMATOLOGY REFERRALS. McCrum C.; Zeljkovic B.; Perera C. Rheumatology (United Kingdom) / 2023;62(Supplement 2):ii45-ii46 DOI: [10.1093/rheumatology/kead104.091](https://dx.doi.org/10.1093/rheumatology/kead104.091)
25. The role of a physiotherapy joint injection clinic within a rheumatology department. Griffiths A.; Lynch M.; Clewes A.; Dawson J.; Abernethy R. Rheumatology / 2009;48(SUPPL. 1):i149 DOI: [10.1093/rheumatology/kep740](https://dx.doi.org/10.1093/rheumatology/kep740)
26. DEVELOPMENT AND EVALUATION OF AN ADVANCED PRACTICE PHYSIOTHERAPIST-LED NEW PATIENT ASSESSMENT SERVICE IN AN AUSTRALIAN RHEUMATOLOGY OUTPATIENT SETTING. Wigg A.; Milanese S.; Ayres A.; Black R.; Limaye V.; Proudman S. Internal Medicine Journal / 2023;53(Supplement 1):14 DOI: [10.1111/imj.16057](https://dx.doi.org/10.1111/imj.16057)
27. Independent prescriber physiotherapist led balance clinic: the Southport and Ormskirk pathway.. BURROWS, L.; LESSER, T. H.; KASBEKAR, A. V.; ROLAND, N.; BILLING, M. Journal of Laryngology & Otology 05// 2017;131(5):417-424 DOI: [10.1017/S0022215117000342](https://dx.doi.org/10.1017/S0022215117000342)
28. Benefit of new pathway involving community neuro-physiotherapist piloting neurology assessment service (NAS) for patient referred by GP to neurology service. Nadar Arulmani G. Physiotherapy (United Kingdom) / 2021;113(Supplement 1):e116-e117 DOI: [10.1016/j.physio.2021.10.100](https://dx.doi.org/10.1016/j.physio.2021.10.100)
29. Implementation of non-medical prescribing (NMP) within the clinical CF setting. Henry A.C.; Forster E.; Bell N.J.; Bateman K. Journal of Cystic Fibrosis / 2014;13(SUPPL. 2):S90
30. Implementation of an expanded-scope-of-practice physiotherapist role in a regional hospital emergency department. Goodman, Donna; Harvey, Desley; Cavanagh, Tania; Nieman, Rebecca. Rural and remote health / 2018;18(2):4212 DOI: [10.22605/RRH4212](https://dx.doi.org/10.22605/RRH4212)
31. Was the impact of COVID-19 on a spinal triage service as significant as expected? A retrospective service evaluation: Results and evaluation. Wood, Lianne; Eveleigh, Carla; Dixon, Matthew; Dunstan, Eleanor; Salem, Khalid. Musculoskeletal care / 2022;20(3):697-704 DOI: [10.1002/msc.1680](https://dx.doi.org/10.1002/msc.1680)
32. Development of a physiotherapy-led atraumatic back pain pathway: A novel initiative to improve the management of complex back pain in the emergency village. Angus M.L.; Martin B.; Dickens V.; Mohammad S.; Siddique I. BMJ Innovations / 2020;6(4):233-238 DOI: [10.1136/bmjinnov-2019-000366](https://dx.doi.org/10.1136/bmjinnov-2019-000366)
33. A review of the surgical conversion rate and independent management of spinal extended scope practitioners in a secondary care setting. Wood, L; Hendrick, P; Boszczyk, B; Dunstan, E. Annals of the Royal College of Surgeons of England / 2016;98(3):187-91 DOI: [10.1308/rcsann.2016.0054](https://dx.doi.org/10.1308/rcsann.2016.0054)
34. 5 Year evaluation of a physio led Virtual Fracture Clinic. Lyle V. Physiotherapy (United Kingdom) / 2022;114(Supplement 1):e118-e119 DOI: [10.1016/j.physio.2021.12.070](https://dx.doi.org/10.1016/j.physio.2021.12.070)
35. Development, implementation and evaluation of an advanced practice in continence and women's health physiotherapy model of care. Brennen, Robyn; Sherburn, Margaret; Rosamilia, Anna. The Australian & New Zealand journal of obstetrics & gynaecology / 2019;59(3):450-456 DOI: [10.1111/ajo.12974](https://dx.doi.org/10.1111/ajo.12974)
36. Back off Specialists. Mawson, Barbara Annette. International Journal of Integrated Care (IJIC) Supplement1 2018;18():1-2 DOI: [10.5334/ijic.s1002](https://dx.doi.org/10.5334/ijic.s1002)
37. Physiotherapy led musculoskeletal interface (MSKI) team evaluation of patient experience, practice and evaluation of discharge outcomes. Candy E.A.; Haworth-Booth S. Physiotherapy (United Kingdom) / 2015;101(SUPPL. 1):eS197 DOI: [10.1016/j.physio.2015.03.360](https://dx.doi.org/10.1016/j.physio.2015.03.360)
38. The efficacy of advanced practice physiotherapy assessment for cervical and lumbar spine pathologies. Motyka E.; Banaszek D.; Inglis T.; Street J. CMAJ. Canadian Medical Association Journal / 2019;62(4 Supplement 1):S55 DOI: [10.1503/cjs.010919](https://dx.doi.org/10.1503/cjs.010919)
39. Data analysis on the role of the Independent Prescriber in Physiotherapy led spasticity clinics. Curran J. Physiotherapy (United Kingdom) / 2021;113(Supplement 1):e121-e122 DOI: [10.1016/j.physio.2021.10.107](https://dx.doi.org/10.1016/j.physio.2021.10.107)
40. Improving the Standard of Care for People with Ankylosing Spondylitis and a New Approach to Developing Specialist ESP-Led AS Clinics. Van Rossen L.; Withrington R.H. Musculoskeletal Care / 2012;10(3):171-177 DOI: [10.1002/msc.1015](https://dx.doi.org/10.1002/msc.1015)
41. Evaluation of the First Contact Physiotherapy (FCP) model of primary care: patient characteristics and outcomes. Stynes, S; Jordan, K P; Hill, J C; Wynne-Jones, G; Cottrell, E; Foster, N E; Goodwin, R; Bishop, A. Physiotherapy / 2021;113(p8c, 0401223):199-208 DOI: [10.1016/j.physio.2021.08.002](https://dx.doi.org/10.1016/j.physio.2021.08.002)
42. The St. Vincent's university hospital (SVUH) physiotherapist-led back pain screening clinic: An audit of patient management. Caffrey A.; Dudeney S.; Fitzgerald O.; Mc Loughlin C.; Daly O. Physiotherapy (United Kingdom) / 2011;97(SUPPL. 1):eS1446-eS1447 DOI: [10.1016/j.physio.2011.04.003](https://dx.doi.org/10.1016/j.physio.2011.04.003)
43. Physiotherapy first -- improving urogynaecology patient flow and clinical outcomes. Nucifora, J.; Bongers, M.; Howard, Z.; Jackman, A.; Corcoran, K.; Weir, K.; Briffa, K. Australian & New Zealand Continence Journal //Spring2018 2018;24(3):90-91
44. Patients Are Satisfied with Advanced Practice Physiotherapists in a Role Traditionally Performed by Orthopaedic Surgeons Commentary. Sawka, C PHYSIOTHERAPY CANADA 2010;62(4):306-307 DOI: [10.3138/physio.62.4.306](https://dx.doi.org/10.3138/physio.62.4.306)
45. Our experience of employing a musculoskeletal practitioner in general practice. Leach, Jack; Lievesley, Kurt. British Journal of General Practice 03// 2020;70(692):110-110 DOI: [10.3399/bjgp20X708413](https://dx.doi.org/10.3399/bjgp20X708413)
46. The utility of esp triage in a specialist secondary care spinal centre: A service evaluation. Dunstan E.; Wood L. Manual Therapy / 2016;25():e115-e116 DOI: [10.1016/j.math.2016.05.209](https://dx.doi.org/10.1016/j.math.2016.05.209)
47. Physical Therapy, Occupational Therapy, and Speech Language Pathology in the Emergency Department: Specialty Consult Services to Enhance the Care of Older Adults. Pontius, Elizabeth A; Anderson, Robert S Jr. Emergency medicine clinics of North America / 2021;39(2):419-427 DOI: [10.1016/j.emc.2021.01.005](https://dx.doi.org/10.1016/j.emc.2021.01.005)
48. Patient view of the advanced practitioner (AP) role in primary care: A realist-informed synthesis. Morris, Leah; Moule, Pam; Pearson, Jennifer; Foster, Dave; Walsh, Nicola. Musculoskeletal care / 2021;19(4):462-472 DOI: [10.1002/msc.1554](https://dx.doi.org/10.1002/msc.1554)
49. Virtual Acute Shoulder Clinic (VASC): A Safe, Sustainable and Cost-Effective Model. Al-Abbasi, Ghaith; Taylor, Gemma; Tanagho, Andy. Indian Journal of Orthopaedics 06// 2025;59(6):793-799. New York, New York Springer Nature 2025 06// DOI:10.1007/s43465-024-01311-3
50. WHAT MATTERS TO PATIENTS? PATIENTS' EXPERIENCES, BARRIERS, ENABLERS AND ACCEPTABILITY OF ATTENDING ADVANCED PRACTICE PHYSIOTHERAPY ASSESSMENTS AND PHYSIOTHERAPY-LED OSTEOARTHRITIS CARE IN COMMUNITY SETTINGS. Gibbs, A; Ezzat, AM; Wallis, J; Kemp, JL; Taylor, N; Low, J; Barton, CJ. OSTEOARTHRITIS AND CARTILAGE 2025;33

**Wrong intervention**

1. Patient satisfaction with ACPAC program trained extended role practitioners: A multi-centre study. Warmington K.; Kennedy C.; Lineker S.; Soever L.; Passalent L.; Lundon K.; Shupak R.; Schneider R. Journal of Rheumatology / 2012;39(8):1747-1748 DOI: [10.3899/jrheum.120615](https://dx.doi.org/10.3899/jrheum.120615)
2. Role substitution of specialist medical doctors with allied-health professionals: A qualitative exploration of patients' experiences and perceptions of healthcare quality. Mutsekwa, RN; Byrnes, JM; Larkins, V; Canavan, R; Angus, RL; Campbell, KL JOURNAL OF EVALUATION IN CLINICAL PRACTICE 2022;28(6):1096-1105 DOI: [10.1111/jep.13691](https://dx.doi.org/10.1111/jep.13691)
3. A combined orthopaedic and physiotherapy service for patients with spinal disorders: A patient satisfaction index outcome. Chandorkar P.; Setiobudi T.; Rahizan; Cong C.C.; Lee C.L.; Koh A.H.; Tan A.; Teoh N.; Tan H.Y. Annals of the Academy of Medicine Singapore / 2014;43(9 SUPPL. 1):S73
4. Patient perspectives of general practice consultation for musculoskeletal disorders: A qualitative study. Thomas, Rachel; Berry, Alice; Cramp, Fiona; Walsh, Nicola. Musculoskeletal care / 2024;22(2):e1904 DOI: [10.1002/msc.1904](https://dx.doi.org/10.1002/msc.1904)
5. Extended roles in primary care when physiotherapist-initiated referral to X-ray can save time and reduce costs. Peterson, Gunnel; PortstrOm, Marie; Frick, Jens. International journal for quality in health care : journal of the International Society for Quality in Health Care / 2021;33(3): DOI: [10.1093/intqhc/mzab122](https://dx.doi.org/10.1093/intqhc/mzab122)
6. Patient satisfaction with a new healthcare provider: Advanced clinician practitioner in arthritis care program-trained clinicians. Warmington K.; Kennedy C.; Lineker S.C.; Soever L.J.; Passalent L.A.; Lundon K.; Shupak R. Arthritis and Rheumatism / 2011;63(10 SUPPL. 1)
7. Can a physiotherapy student assume the role of an advanced practice physiotherapist in Orthopaedic surgery triage? A prospective observational study. Yin, David; Cabana, Francois; Tousignant-Laflamme, Yannick; Bedard, Sonia; Tousignant, Michel. BMC musculoskeletal disorders / 2019;20(1):498 DOI: [10.1186/s12891-019-2864-x](https://dx.doi.org/10.1186/s12891-019-2864-x)
8. Expanded role for the physical therapist. Screening musculoskeletal disorders. James, J J; Stuart, R B. Physical therapy / 1975;55(2):121-31
9. Ontario Inter-professional Spine Assessment and Education Clinics (ISAEC): Patient, Provider and System Impact of an Integrated Model of Care for the Management of Low Back Pain (LBP)...First North American Conference on Integrated Care, October 4-7, 2021. Rampersaud, Raja. International Journal of Integrated Care (IJIC) ;22():1-2 DOI: [10.5334/ijic.ICIC21174](https://dx.doi.org/10.5334/ijic.ICIC21174)
10. Patients with spondyloarthritis are equally satisfied with follow-up by physiotherapist and rheumatologist. Holoyen, Pauline Kjelsvik; Stensdotter, Ann-Katrin. Musculoskeletal care / 2018;16(3):388-397 DOI: [10.1002/msc.1241](https://dx.doi.org/10.1002/msc.1241)
11. Patient, Rheumatologist and Therapist Perspectives on the Implementation of an Allied Health Rheumatology Triage (AHRT) Initiative in Ontario Rheumatology Clinics. Fullerton, Laura M; Brooks, Sydney; Sweezie, Raquel; Ahluwalia, Vandana; Bombardier, Claire; Gagliardi, Anna R. Pragmatic and observational research / 2020;11(101688693):1-12 DOI: [10.2147/POR.S213966](https://dx.doi.org/10.2147/POR.S213966)
12. Patient satisfaction with care received from advanced clinician practitioner in arthritis care (ACPAC) program-trained practitioners. Warmington K.; Kennedy C.; Lineker S.; Soever L.; Passalent L.; Lundon K.; Shupak R.; Schneider R. Physiotherapy (United Kingdom) / 2011;97(SUPPL. 1):eS1328-eS1329 DOI: [10.1016/j.physio.2011.04.002](https://dx.doi.org/10.1016/j.physio.2011.04.002)
13. The patient perspective: arthritis care provided by Advanced Clinician Practitioner in Arthritis Care program-trained clinicians. Warmington, Kelly; Kennedy, Carol A; Lundon, Katie; Soever, Leslie J; Brooks, Sydney C; Passalent, Laura A; Shupak, Rachel; Schneider, Rayfel. Open access rheumatology : research and reviews / 2015;7(101688698):45-53
14. Multi-site evaluation of advanced practice hand therapy clinics for the management of patients with trigger digit. Burton, Christopher; Palmer, Michelle A; Fanton, Lauren; Cox, Ruth; Wishart, Laurelie R. Journal of hand therapy : official journal of the American Society of Hand Therapists / 2022;35(4):655-664 DOI: [10.1016/j.jht.2021.06.007](https://dx.doi.org/10.1016/j.jht.2021.06.007)

**Inadequate or Incomplete data**

1. Good perceived quality of physiotherapy triage assessment of patients referred for orthopaedic consultation. Samsson K.S.; Larsson M.E.H. Physiotherapy (United Kingdom) / 2015;101(SUPPL. 1):eS1331-eS1332 DOI: [10.1016/j.physio.2015.03.1260](https://dx.doi.org/10.1016/j.physio.2015.03.1260)
2. Advanced practice physiotherapy services: Qualitative study of patient experiences. Fennelly O.; Blake C.; Fitz Gerald O.; Breen R.; Caffrey A.; Smart K.; Fletcher L.; Corcoran S.; Casserly Feeney S.; Ni She E.; Cunningham C. Osteoporosis International / 2018;29(1 Supplement 1):S198 DOI: [10.1007/s00198-018-4465-1](https://dx.doi.org/10.1007/s00198-018-4465-1)
3. Utilizing a novel model of care to improve wait-times and diagnosis of patients with upper-extremity pain...First North American Conference on Integrated Care, October 4-7, 2021, Toronto, Ontario Hawke, Christopher; Veillette, Christian; Leroux, Timothy; Soever, Leslie International Journal of Integrated Care (IJIC) ;22():1-2 DOI: [10.5334/ijic.ICIC21298](https://dx.doi.org/10.5334/ijic.ICIC21298)
4. P67. The efficacy of advanced practice physiotherapy assessment for cervical and lumbar spine pathologies. Banaszek D.; Inglis T.; Ailon T.; Charest-Morin R.; Dea N.; Fisher C.G.; Kwon B.K.; Paquette S.J.; Street J. Spine Journal / 2019;19(9 Supplement):S189 DOI: [10.1016/j.spinee.2019.05.491](https://dx.doi.org/10.1016/j.spinee.2019.05.491)
5. The extended role of a physiotherapist in an out-patient orthopaedic clinic. Hockin J.; Bannister G. Physiotherapy / 1994;80(5):281-284 DOI: 10.1016/S0031-9406(10)61050-4
6. A pilot study to investigate the impact of physiotherapy services for patients presenting with musculoskeletal conditions in an emergency department setting in Singapore. Ngo X.; Foo C.L.; Yee Y.W.; Gan Y.M.; Mohamed H.; Anuar K.; Loh W.B.; Gadru R.; Udipi S. Annals of the Academy of Medicine Singapore / 2016;45(9 Supplement 1):S374
7. HIGH PATIENT SATISFACTION WITH AN MDT ADVANCED PRACTICE PHYSIOTHERAPIST AND NURSE CATEGORY 3 TRIAGE AND ASSESSMENT CLINIC. McCrum C.; Zeljkovic B.; Perera C. Internal Medicine Journal / 2022;52(SUPPL 3):44 DOI: [10.1111/imj.15756](https://dx.doi.org/10.1111/imj.15756)
8. Advanced practice continence & women's health physiotherapy in urogynaecology. Hraetz H. International Urogynecology Journal / 2018;29(Supplement 1):S159 DOI: [10.1007/s00192-018-3752-x](https://dx.doi.org/10.1007/s00192-018-3752-x)
9. Physiotherapy as a first point of contact in general practice: a solution to a growing problem?. Goodwin, Rob W; Hendrick, Paul A. Primary health care research & development / 2016;17(5):489-502 DOI: 10.1017/S1463423616000189

**Wrong outcome**

1. Acceptability of Physiotherapists in the Emergency Department for the Care of Adults With Musculoskeletal Disorders: An Exploratory Survey of Patient' Perspective. Beland A.; Matifat E.; Cournoyer E.; Perreault K.; Desmeules F. Journal of Acute Care Physical Therapy / 2022;13(2):83-91 DOI: [10.1097/JAT.0000000000000178](https://dx.doi.org/10.1097/JAT.0000000000000178)
2. Patient acceptability of the physiotherapy first contact practitioner role in primary care: A realist informed qualitative study. Morris, Leah; Moule, Pam; Pearson, Jennifer; Foster, Dave; Walsh, Nicola Musculoskeletal care / 2021;19(1):38-51 DOI: [10.1002/msc.1505](https://dx.doi.org/10.1002/msc.1505)
3. Perceptions of advanced clinician practitioner in arthritis care (ACPAC) program-trained practitioners: Roles and role utilization within the Ontario healthcare system. Warmington K.; Kennedy C.; Lundon K.; Shupak R.; Rozmovits L.; Lineker S.; Schneider R. Journal of Rheumatology / 2011;38(6):1174-1175 DOI: 10.3899/jrheum.110506
4. Exploration of patients' perspectives of quality within an extended scope physiotherapists' spinal screening service. Reeve, Sarah; May, Stephen. Physiotherapy theory and practice / 2009;25(8):533-43 DOI: [10.3109/09593980802664869](https://dx.doi.org/10.3109/09593980802664869)
5. Transforaminal epidural steroid injection in lumbar spinal stenosis: an observational study with two-year follow-up. Davis, Niel; Hourigan, Patrick; Clarke, Andrew. British journal of neurosurgery / 2017;31(2):205-208 DOI: 10.1080/02688697.2016.1206188
6. The effectiveness of orthopaedic triage by extended scope physiotherapists. Hattam P. Clinical Governance / 2004;9(4):244-252 DOI: [10.1108/14777270410566661](https://dx.doi.org/10.1108/14777270410566661)
7. The experiences of patients with musculoskeletal conditions accessing first contact physiotherapy practitioner appointments in general practice in the UK: A qualitative study. Lamb, Kirsten; Comer, Christine; Walsh, Nicola; Smith, Julia; Tang, Krystal; McHugh, Gretl. Musculoskeletal care / 2024;22(2):e1908 DOI: [10.1002/msc.1908](https://dx.doi.org/10.1002/msc.1908)
