# Supplementary material for: Patient satisfaction with advanced practice physiotherapy internationally: A systematic mixed studies review
Source: PLoS One. 2026 Feb 11;21(2):e0342674. doi: 10.1371/journal.pone.0342674 (PMC12893546; doi:10.1371/journal.pone.0342674)
Supplement: S6 File — (DOCX) [file pone.0342674.s006.docx]

**S6 File. Quality Assessment**

| **First author  (year)** | **Methodological quality criteria** | | | | | **%** | **MMAT Quality appraisal** |
| --- | --- | --- | --- | --- | --- | --- | --- |
|  | **Study design category: Qualitative** | | | | |  |  |
|  | 1.1. Is the qualitative approach appropriate to answer the research question? | 1.2. Are the qualitative data collection methods adequate to address the research question? | 1.3. Are the findings adequately derived from the data? | 1.4. Is the interpretation of results sufficiently substantiated by data? | 1.5. Is there coherence between qualitative data sources, collection, analysis and interpretation? |  |  |
| Fennelly, O (2020) | Y | Y | Y | Y | Y | 100 | High |
| Gillis, K (2014) | Y | Y | Y | Y | Y | 100 | High |
| Goodwin, R (2021) | CT | CT | Y | Y | N | 40 | Low |
| Harding, P (2015) | Y | Y | Y | Y | Y | 100 | High |
| Vader, K (2022) | Y | Y | Y | Y | Y | 100 | High |
| Wood, L (2022) | Y | Y | Y | Y | CT | 80 | Moderate |
| Blondin, J (2024) | Y | Y | Y | Y | Y | 100 | High |
| Kechichian, A (2024) | Y | Y | Y | Y | Y | 100 | High |
| Lafrance, S (2024) | CT | Y | Y | Y | Y | 80 | Moderate |
|  |  |  |  |  |  |  |  |
|  | **Study design category: Randomized controlled trial** | | | | |  |  |
|  | 2.1. Is randomization appropriately performed? | 2.2. Are the groups comparable at baseline? | 2.3. Are there complete outcome data? | 2.4. Are outcome assessors blinded to the intervention provided? | 2.5 Did the participants adhere to the assigned intervention? |  |  |
| Daker-White, G (1999) | Y | Y | Y | N | Y | 80 | Moderate |
| Gustavsson, L (2023) | Y | Y | N | N | Y | 60 | Moderate |
| Marks, D (2016) | Y | Y | Y | Y | Y | 100 | High |
| Samsson, KS (2016) | Y | Y | N | N | Y | 60 | Moderate |
| Lafrance, S (2024) | Y | Y | CT | N | N | 40 | Low |
|  |  |  |  |  |  |  |  |
|  | **Study design category: Quantitative non-randomized** | | | | |  |  |
|  | 3.1. Are the participants representative of the target population? | 3.2. Are measurements appropriate regarding both the outcome and intervention (or exposure)? | 3.3. Are there complete outcome data? | 3.4. Are the confounders accounted for in the design and analysis? | 3.5. During the study period, is the intervention administered (or exposure occurred) as intended? |  |  |
| Desmeules, F (2013) | Y | CT | Y | N | Y | 60 | Moderate |
| Kennedy, DM (2010) | Y | Y | Y | N | Y | 80 | Moderate |
| Lowry, V (2020) | Y | N | Y | CT | Y | 60 | Moderate |
| Matifat, E (2019) | Y | Y | N | N | Y | 60 | Moderate |
| McClellan, CM (2006) | CT | Y | N | CT | Y | 40 | Low |
| Murphy, MT (2021) | CT | Y | Y | Y | Y | 80 | Moderate |
| Razmjou, H (2013) | CT | Y | Y | CT | CT | 40 | Low |
| Robarts, S (2017) | Y | Y | Y | CT | Y | 80 | Moderate |
| Schulz, P (2016) | Y | Y | Y | N | Y | 80 | Moderate |
| Taylor, NF (2011) | N | Y | Y | Y | Y | 80 | Moderate |
| Truter, P (2024) | CT | Y | N | CT | Y | 40 | Low |
|  |  |  |  |  |  |  |  |
|  | **Study design category: Quantitative descriptive** | | | | |  |  |
|  | 4.1. Is the sampling strategy relevant to address the research question? | 4.2. Is the sample representative of the target population? | 4.3. Are the measurements appropriate? | 4.4. Is the risk of nonresponse bias low? | 4.5. Is the statistical analysis appropriate to answer the research question? |  |  |
| Downie, F (2019) | CT | CT | N | N | Y | 20 | Low |
| Resteghini, P (2003) | CT | N | CT | CT | Y | 20 | Low |
| Matifat, E (2025) | N | Y | Y | N | Y | 60 | Moderate |
|  |  |  |  |  |  |  |  |
|  | **Study design category: Mixed methods** | | | | |  |  |
|  | 5.1. Is there an adequate rationale for using a mixed methods design to address the research question? | 5.2. Are the different components of the study effectively integrated to answer the research question? | 5.3. Are the outputs of the integration of qualitative and quantitative components adequately interpreted? | 5.4. Are divergences and inconsistencies between quantitative and qualitative results adequately addressed? | 5.5. Do the different components of the study adhere to the quality criteria of each tradition of the methods involved? |  |  |
| Carey, N (2020) | N | Y | N | Y | N | 40 | Low |
| Bødskov E B (2022) | Y | Y | Y | CT | Y | 80 | Moderate |
| Gibbs, AJ (2020) | Y | Y | Y | Y | Y | 100 | High |
| Harding, P (2018) | N | Y | N | Y | N | 40 | Low |
| Morris, J (2015) | N | N | Y | N | CT | 20 | Low |
| Soever, L (2023) | N | N | Y | Y | N | 40 | Low |
| Booth, R (2019) | Y | Y | Y | Y | CT | 80 | Moderate |
|  |  |  |  |  |  |  |  |
| **Thresholds** | **%** |  |  |  |  |  |  |
| Low | 0-40 |  |  |  |  |  |  |
| Mod | 60-80 |  |  |  |  |  |  |
| High | 100 |  |  |  |  |  |  |
